# Selective syntheses of leuconolam, leuconoxine, and mersicarpine alkaloids from a common intermediate through regiocontrolled cyclizations by Staudinger reactions†
†Electronic supplementary information (ESI) available: Experimental details and procedures, compound characterization data, copies of ^1^H and ^13^C NMR spectra for new compounds. See DOI: 10.1039/c4qo00312h



**DOI:** 10.1039/c4qo00312h

**Published:** 2015-01-27

**Authors:** Zining Li, Qian Geng, Zhe Lv, Beau P. Pritchett, Katsuaki Baba, Yoshitaka Numajiri, Brian M. Stoltz, Guangxin Liang

**Affiliations:** a State Key Laboratory and Institute of Elemento-organic Chemistry , Collaborative Innovation Center of Chemical Science and Engineering (Tianjin) , Nankai University , Tianjin 300071 , China . Email: lianggx@nankai.edu.cn; b The Warren and Katharine Schlinger Laboratory of Chemistry and Chemical Engineering , Division of Chemistry and Chemical Engineering , California Institute of Technology , Pasadena , California 91125 , USA . Email: stoltz@caltech.edu

## Abstract

Selective syntheses of leuconolam, leuconoxine, and mersicarpine alkaloids bearing distinctive core structures were achieved through Staudinger reactions using a common intermediate. In the key cyclization step, water functioned like a switch to control which core structure to produce. The chemistry allowed for selective syntheses of the group of alkaloids from a simple intermediate through straightforward chemical operations.

## Abstract 2

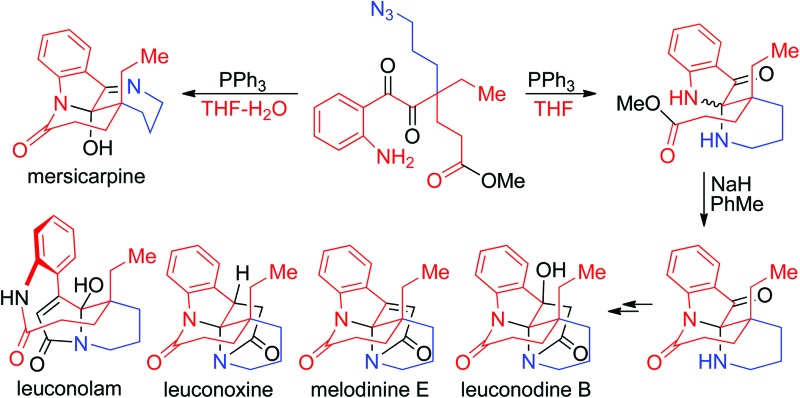
Selective syntheses of alkaloids bearing distinct core structures were enabled by chemically programmed polycyclizations using water as a switch.

## Introduction

**Fig. 1:**
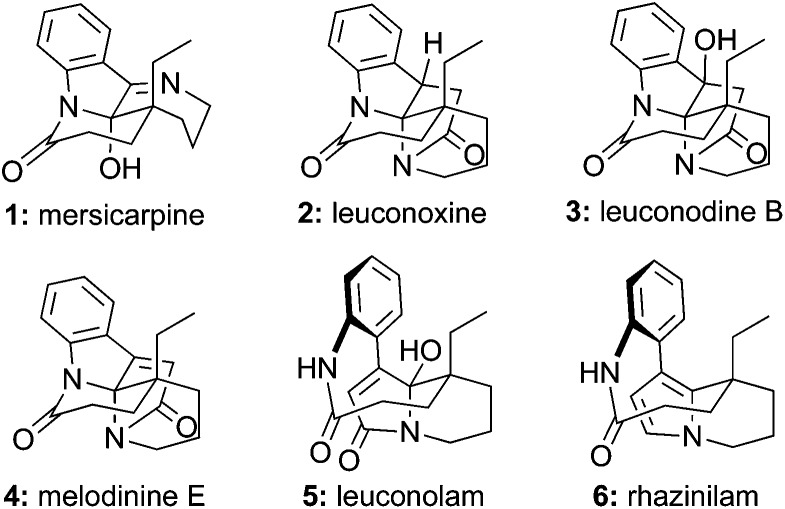
Representative biosynthetically related monoterpene indole alkaloids with distinctive skeleton diversity.

Leuconolam, leuconoxine, and mersicarpine alkaloids showcase the incredible structural diversity of natural products. These monoterpene indole alkaloid families, though sharing the same biogenetic origin,^1^ present distinctive skeletons with three completely different polycyclic patterns (**1–6**, Fig. 1).

Mersicarpine (**1**), isolated from both *Kopsia* and *Leuconotis* species of plants by Kam and co-workers,^2^ features a seven-membered cyclic imine, a δ-lactam, and an all-carbon quaternary center around a fully substituted hemiaminal stereogenic center. Although leuconoxine (**2**),^3^ leuconodine B (**3**),^4^ and melodinine E (**4**)^5^ hold the same δ-lactam and indoline moiety as mersicarpine, different bond connections and two additional carbons create an entirely new skeleton distinguished by an aminal functionality, a piperidine ring, and an extra γ-lactam. Leuconolam (**5**)^6^ and rhazinilam (**6**)^7^ possess an unusual nine-membered lactam and a pyrrole derived-unit. It is proposed that leuconolam is a biosynthetic precusor of melodinine E, which further produces mersicarpine *via* a skeletal rearrangement and subsequent loss of two carbons in the form of acetic acid.^2*a*^ The intriguing structural features and biosynthetic connections of these alkaloids make them appealing synthetic targets.^8^ To date, eight syntheses of mersicarpine^9^ and three syntheses of leuconoxine-type alkaloids have been reported.^9e,g,10^ Leuconolam has been accessed through both total synthesis^9e,g,11^ and oxidative conversion from rhazinilam.^12^ Rhazinilam has been the focus of numerous synthetic efforts.^9g,13^

**Scheme 1:**
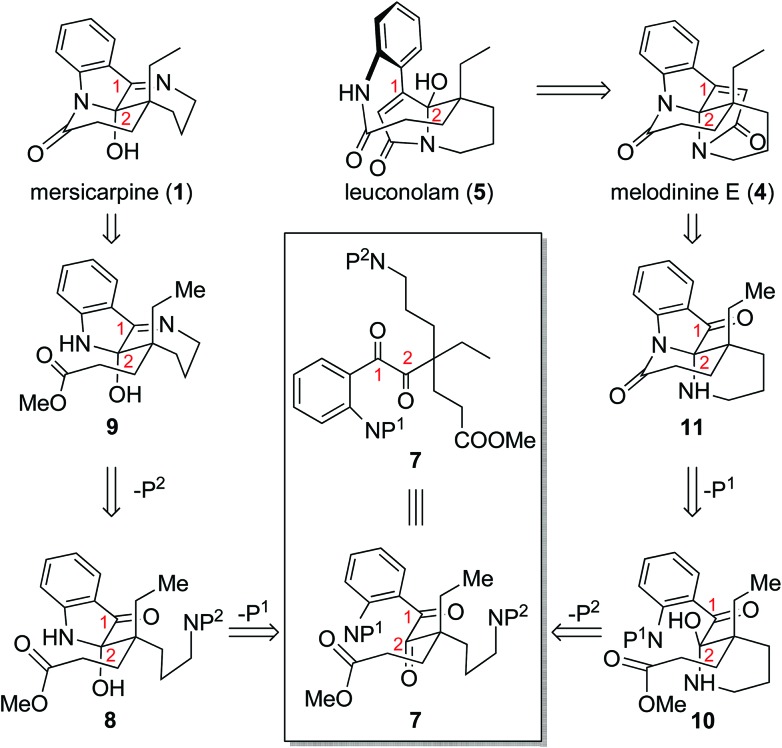
Initial synthetic design of different cyclization sequences leading to distinct molecular skeletons.

Throughout our efforts toward the total synthesis of mersicarpine,^9f,14^ we became increasingly interested in its connections with leuconolam and leuconoxine alkaloids. We envisioned rapid access to all three different polycyclic patterns through a versatile intermediate **7** (Scheme 1). Leuconolam (**5**) could be obtained through disconnection of the C–N bond of melodinine E (**4**). Melodinine E could be accessed from **11** by an acetylation and aldol condensation sequence. Given that mersicarpine (**1**) and **11** have the same oxidation state but different bond connections, we conceived that both compounds could be prepared from a common acyclic intermediate **7** through divergent cyclization sequences. We aimed to take advantage of orthogonal protecting groups P^1^ and P^2^ on the aniline and amine nitrogens, respectively. Upon the removal of P^1^, facile hemiaminal formation at the C2 position would afford **8**, which could in turn produce compound **9** upon removal of P^2^. If instead P^2^ is removed first, a more favourable 6-membered hemiaminal formation would generate intermediate **10**, which could produce compound **11** following P^1^ removal and subsequent lactam formation. It is worth noting that Zhu and co-workers applied a similar strategy in their recent syntheses of these alkaloids, in which they used fine-tuned hydrogenation conditions to control the cyclization sequences.^9*e*^ Herein, we report a new approach to three different classes of alkaloids using Staudinger reaction as a key ring formation step from a common acyclic intermediate.

## Results and discussion

**Scheme 2:**
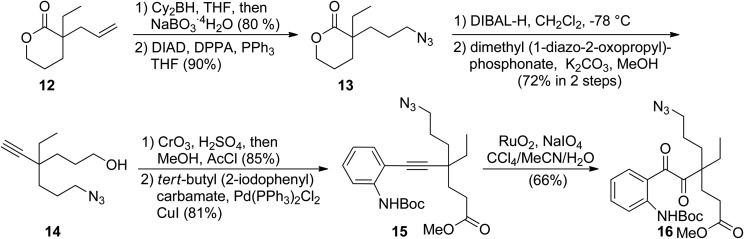
Preparation of the common intermediate **16**.

In the forward synthesis, we chose compound **16** (Scheme 2) to be a preferred intermediate with a Boc-protected aniline (NP^1^) and an azide (NP^2^) as a masked amine to enable differentiable deprotection. Our quick construction of **16** commenced from a known compound **12**.^15^ Hydroboration-oxidation,^16^ followed by a Mitsunobu reaction using DPPA^17^ converted **12** to compound **13** featuring a primary azide. Lactol formation was effected with DIBAL-H, followed by Ohira–Bestmann homologation^18^ afforded alkyne **14**. Oxidation of the primary alcohol, followed by Fischer esterification provided the desired coupling partner for Sonogashira coupling^19^ with *tert*-butyl-(2-iodophenyl) carbamate to furnish **15** in good yield. Ruthenium-catalysed oxidation^20^ of the alkyne afforded 1,2-diketone **16** in 66% yield.

**Scheme 3:**
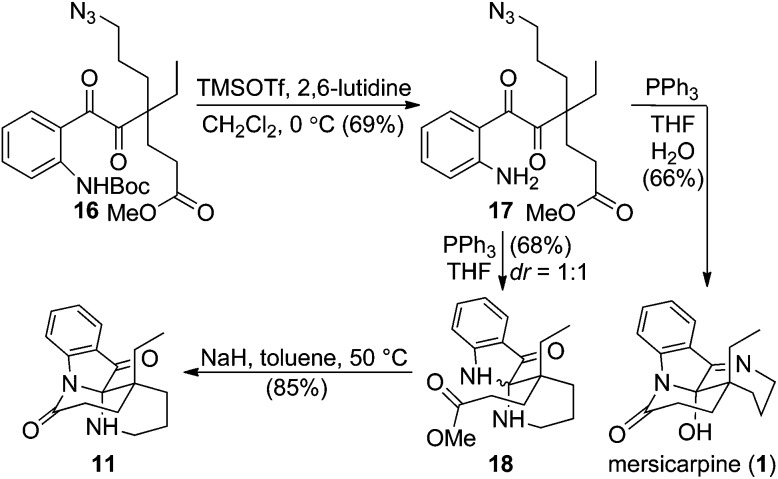
Selective syntheses of mersicarpine and the core structure **11** in leuconoxine-type alkaloids.

We then turned our attention to exploring divergent cyclization sequences involving 1,2-diketone **16** (Scheme 3). To our surprise, compound **17** didn't undergo spontaneous hemiaminal formation, but was isolated in 69% yield following selective removal of the Boc group in **16** with TMSOTf in the presence of 2,6-lutidine.^21^ However, treatment of **17** with triphenylphosphine in a mixed solvent of THF and water cleanly furnished mersicarpine in 66% yield. This remarkably simple reaction forms the three remaining rings in mersicarpine under mild conditions. Notably, the oxidation states of diketone in **17** were exploited to rapidly arrive at the target in a redox-free manner. Importantly, a Staudinger reaction in the absence of water gave an inseparable diastereomeric mixture of compound **18**, which possesses a totally different polycyclic framework. We hypothesize that an aza-Wittig pathway is operative in the absence of water.^22^ In the event, the more favourable 6-membered imine is formed, followed by aminal formation with no facial selectivity. Impressively, when a diastereomeric mixture of **18** was treated with sodium hydride in toluene at 50 °C, compound **11** was generated in 85% yield. This finding indicates that an interconversion of the two diastereomeric aminals formed under the reaction conditions funnels the mixture toward a thermodynamically favored product (**11**).

**Scheme 4:**
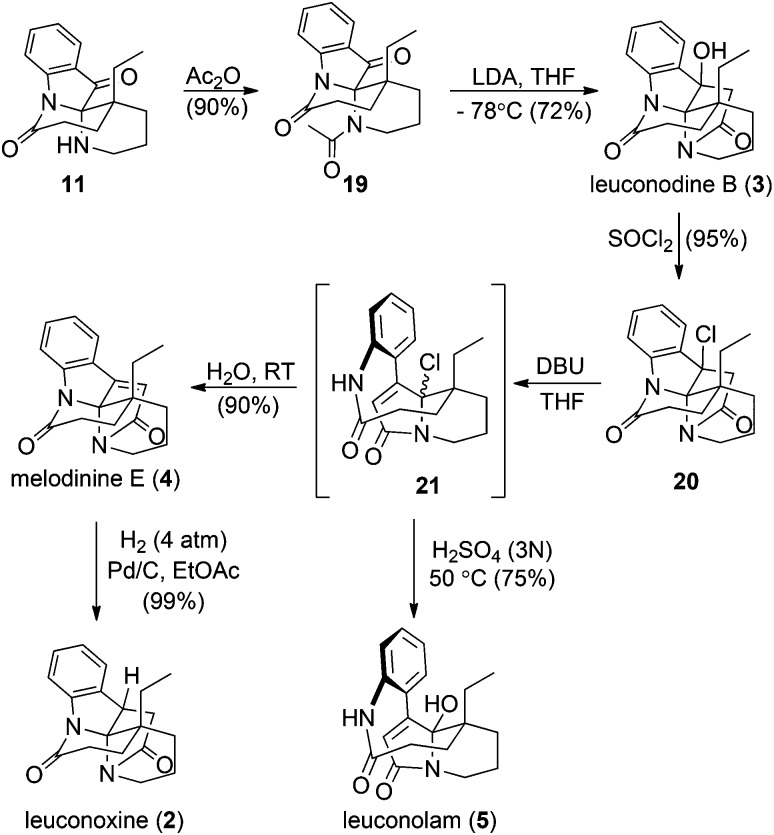
Syntheses of leuconodine B, melodinine E, leuconoxine, and leuconolam.

Key intermediate **11** facilitated completion of the total syntheses of three leuconoxine-type alkaloids as well as leuconolam (Scheme 4). Acetylation of the free amine in the piperidine ring in **11** proceeded smoothly in neat acetic anhydride at room temperature to afford **19**. When treated with LDA at –78 °C, **19** produced leuconodine B readily in 72% yield. The transformation of leuconodine B to melodinine E was fulfilled in 90% yield upon treatment with neat thionyl chloride at room temperature followed by elimination with DBU in THF and subsequent aqueous workup. Initially, we believed that treatment of **20** with DBU would generate melodinine E directly, but surprisingly melodinine E was not detected by ^1^H NMR spectroscopy in the crude mixture without an aqueous workup. The major product was too sensitive to be isolated and attempted purification of this compound with column chromatography produced melodinine E. High resolution mass spectrometry data suggest that treatment of **20** with DBU yields the proposed structure **21**. When the sensitive intermediate **21** was stirred in water at room temperature, melodinine E was produced in 90% yield. Interestingly, when **21** was treated with an aqueous solution of 3 N H_2_SO_4_ at 50 °C, leuconolam was generated in 75% yield. Using conditions reported by Zhu and co-workers, leuconolam can also be prepared directly from melodinine E.^9*e*^ Finally, hydrogenation on melodinine E occurred efficiently to generate leuconoxine in nearly quantitative yield.

**Scheme 5:**
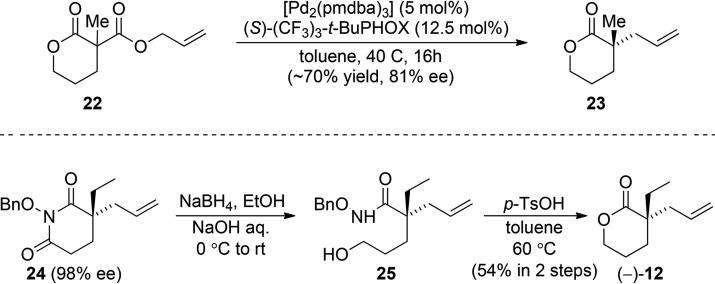
Efforts in preparing optically active **12**.

With efficient racemic syntheses in hand, we took on an effort to produce optically active **12**, thereby achieving formal asymmetric syntheses of these alkaloids (Scheme 5). Initially, we hoped diester **22** could undergo an efficient asymmetric allylic alkylation to construct enantioenriched quaternary lactone **23**. We found that the reaction with diester **22** proceeded smoothly, but with disappointing enantioselectivity (81% ee). Eventually, we were able to generate optically active **12** from an *N*-benzyloxy imide **24**, which could be readily prepared in 80% yield and 98% ee.^23^ Reduction of **24** with an excess of NaBH_4_ formed hydroxamic acid **25** with the desired free primary alcohol. The following acid-induced cyclization of **25** provided the desired lactone (–)-**12** in 54% yield over 2 steps.

## Conclusions

In summary, we have completed total syntheses of mersicarpine (**1**), leuconoxine (**2**), leuconodine (**3**), melodinine E (**4**), and leuconolam (**5**) by controlling specific cyclization sequences through a key Staudinger reaction to access different polycyclic frameworks. Additionally, we have achieved enantioselective formal syntheses of these alkaloids by synthesizing enantioenriched lactone **12** *via* an asymmetric allylic alkylation, reduction, and cyclization sequence.
