# Supplementary material for: Selective syntheses of leuconolam, leuconoxine, and mersicarpine alkaloids from a common intermediate through regiocontrolled cyclizations by Staudinger reactions
Source: Org Chem Front. 2015 Jan 27;2(3):236–40. doi: 10.1039/c4qo00312h (PMC4333676; doi:10.1039/c4qo00312h)

Electronic Supplementary Material (ESI) for Organic Chemistry Frontiers.
This journal is © the Partner Organisations 2015

Supporting Information

Selective Syntheses of Leuconolam, Leuconoxine, and Mersicarpine Alkaloids from a Common Intermediate through Regiocontrolled Cyclizations by Staudinger Reactions

Zining Li, Qian Geng, Zhe Lv, Beau P. Pritchett, Katsuaki Baba, Yoshitaka Numajiri, Brian M. Stoltz* and Guangxin Liang*

State Key Laboratory and Institute of Elemento-organic Chemistry, Collaborative Innovation Center of Chemical Science and Engineering （Tianjin）, Nankai University, Tianjin, China, 300071

lianggx@nankai.edu.cn

The Warren and Katharine Schlinger Laboratory of Chemistry and Chemical Engineering, Division of Chemistry and Chemical Engineering, California Institute of Technology, Pasadena, California 91125

stoltz@caltech.edu

Table of Contents

General information...S-1

Experimental procedures…………………………………………......S-1–S-12

Table of NMR spectroscopic data comparison………..…........S-13–S-17

Reference and Note…...………………........S-18

NMR Spectra..S-19–S-52

HRMS Spectra of Compound **21**...S-53

General information:

All air and moisture sensitive reactions were performed under an atmosphere of argon. Reagents obtained from Acros, Aldrich, J&K, and Aladdin were used without further purification. THF and toluene were dried by distillation over Na/benzophenone. MeCN, CH_2Cl_2, CCl_4 and NEt_3 were dried by distillation over CaH_2, DMF was dried by distillation over CaH_2 under reduced pressure. MeOH was dried by distillation over Mg turnings. TLC inspections were on silica gel GF254 plates. Column chromatography was performed on silica gel (200–300 mesh).

^{1}H NMR and ^{13}C NMR spectra were recorded on a Bruker AVANCE AV400 (400MHz and 100MHz). Signal positions were recorded in ppm with the abbreviations s, d, t, m, and bs denoting singlet, doublet, triplet, multiplet, and broad singlet respectively. All NMR chemical shifts were referenced to residual solvent peaks or to $Si(CH_3)_4$ as an internal standard, spectra recorded in $CDCl_3$ were referenced to residual $CHCl_3$ at 7.26 ppm for ^{1}H NMR or 77.0 ppm for ^{13}C NMR. All coupling constants *J* are quoted in Hz. FTIR spectra were obtained with a Bruker Tensor 27 instrument. All IR samples were prepared as thin film and reported in wave numbers (cm^{-1}). High resolution mass spectra (HRMS) were obtained on an IonSpec QFT mass spectrometer with ESI ionization.

Preparation of compound 13

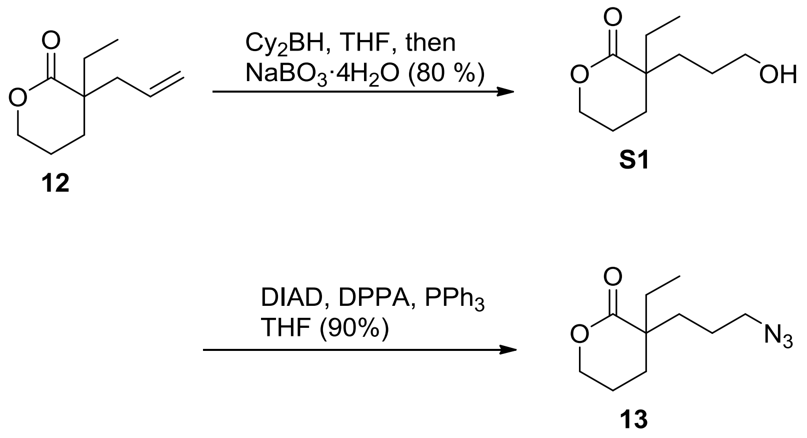

To a solution of BH_3 ·THF (1 M, 0.60 mL, 0.6 mmol, 1.2 equiv) in THF at 0 ℃ was added cyclohexene (98.5 mg, 1.2 mmol, 2.4 equiv) in THF (1 mL). The mixture was stirred at 0 ℃ for 1 h. Then a solution of compound **12** (84 mg, 0.5 mmol, 1 equiv) in THF (1 mL) was added. The mixture was stirred at room temperature for another 1 h. The resulting solution was stirred at room temperature for 5 h after quenched by addition of $NaBO_3 \cdot 4H_2O$ (280 mg, 1.8 mmol, 3.6 equiv) and water (2 mL) at 0 ℃. The reaction mixture was extracted with EtOAc (5×3 mL). The combined solution

was concentrated and purified by column chromatography (2:1 petroleum ether:ethyl acetate) to give **S1** (75 mg, 0.4 mmol, 80%) as a clear oil. Data for **S1**: R_f 0.15 (2:1 petroleum ether:ethyl acetate); ^{1}H NMR (400 MHz, $CDCl_3$) δ 4.30 (t, J = 5.5 Hz, 2 H), 3.66-3.55 (m, 2 H), 1.90-1.82 (m, 2 H), 1.81-1.70 (m, 4 H), 1.64-1.54 (m, 4 H), 0.90 (t, J = 7.4 Hz, 3 H); ^{13}C NMR (100 MHz, $CDCl_3$) δ 176.1, 70.1, 62.7, 45.9, 35.1, 32.2, 29.1, 27.6, 21.3, 8.6; IR (thin film) ν_{max} 3411, 2945, 2878, 1719, 1460, 1399, 1255, 1161; HRMS (ESI) Calcd for $C_{10}H_{19}O_3$ $[M+H]^+$ 187.1329, found: 187.1333.

To a solution of **S1** (18.6 mg, 0.1 mmol, 1 equiv) and PPh_3 (53 mg, 0.2 mmol, 2.0 equiv) in THF (2 mL) was added a solution of DPPA (82 mg, 0.3 mmol, 3.0 equiv) in THF (1 mL) and DIAD (61 mg, 0.3 mmol, 3.0 equiv) at 0 ℃. The reaction mixture was stirred at 0 ℃ for 2 h. The reaction mixture was concentrated and purified by column chromatography (8:1 petroleum ether:ethyl acetate) to give compound **13** (19 mg, 0.09 mmol, 90%) as a clear oil. Data for compound **13**: R_f 0.45 (4:1 petroleum ether:ethyl acetate); ^{1}H NMR (400 MHz, $CDCl_3$) δ 4.31 (t, J = 5.4 Hz, 2 H), 3.34-3.22 (m, 2 H), 1.90-1.84 (m, 2 H), 1.83-1.68 (m, 4 H), 1.65-1.55 (m, 4 H), 0.91 (t, J = 7.5 Hz, 3 H); ^{13}C NMR (100 MHz, $CDCl_3$) δ 175.4, 70.1, 51.6, 45.9, 36.0, 32.0, 29.3, 24.1, 21.2, 8.6; IR (thin film) ν_{max} 2967, 2879, 2096, 1723, 1462, 1252; HRMS (ESI) Calcd for $C_{10}H_{18}N_3O_2$ $[M+H]^+$ 212.1394, found: 212.1396.

Preparation of compound 14

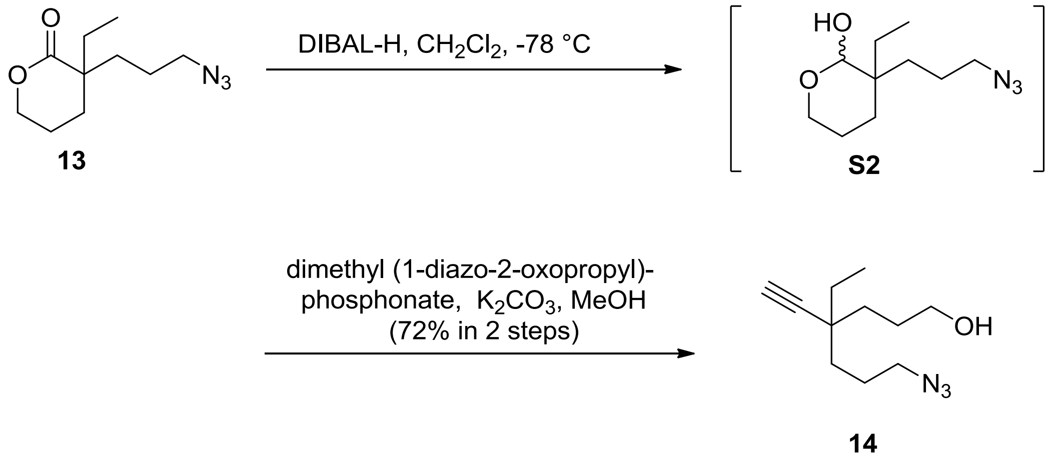

To a solution of compound **13** (21 mg, 0.1 mmol, 1 equiv) in CH_2Cl_2 (2 mL) was added DIBAL-H (1.0 M in toluene, 0.15 mmol, 1.2 equiv) at -78 ℃ slowly. After stirred for 30 min at the same temperature, the mixture was quenched with MeOH (2 mL) carefully. Anhydrous K_2CO_3 (27.2 mg, 0.2 mmol, 2 equiv) and dimethyl-1-diazo-2-oxopropylphosphonate (28.8 mg, 1.5 mmol, 1.5 equiv) was added. The mixture was stirred at room temperture for 8 h. The reaction mixture was diluted with Et_2O (15 mL), washed with water (3×5 mL), dried over anhydrous Na_2SO_4, and filtered. Solvent was removed under reduced pressure to give a light yellow oil. The crude product was purified by column chromatography (8:1 petroleum ether:ethyl

acetate) to give compound **14** (15 mg, 0.072 mmol, 72%) as a clear oil. Data for compound **14**: R_f 0.36 (4:1 petroleum ether:ethyl acetate); ^{1}H NMR (400 MHz, $CDCl_3$) δ 3.66 (t, J = 6.4 Hz, 2 H), 3.29 (t, J = 6.7 Hz, 2 H), 2.12 (s, 1 H), 1.74-1.60 (m, 5 H), 1.54-1.44 (m, 6 H), 0.93 (t, J = 7.4 Hz, 3 H); ^{13}C NMR (100 MHz, $CDCl_3$) δ 89.3, 70.3, 63.1, 51.8, 38.1, 34.7, 33.7, 30.6, 27.6, 24.0, 8.5; IR (thin film) ν_{max} 3369, 3301, 2944, 2876, 2095, 1454, 1258, 1058; HRMS (ESI) Calcd for $C_{11}H_{20}N_3O_3$ $[M+H]^+$ 210.1601, found: 210.1605.

Preparation of compound 15

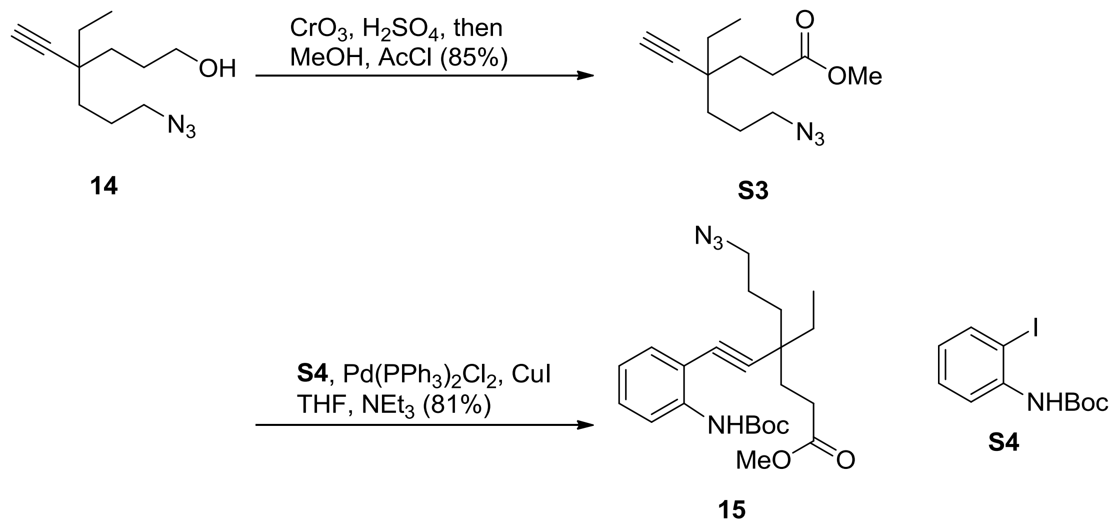

To a solution of compound **14** (209 mg, 1.0 mmol, 1 equiv) in acetone (10 mL) was added a solution of CrO_3 in H_2SO_4 (8 N, about 2 mL) at 0 ℃ dropwise until the solution remained orange. The mixture was stirred for 10 min before water (20 mL) was added. The reaction mixture was extracted with Et_2O (5×5 mL), dried over anhydrous Na_2SO_4, and filtered. Solvent was removed under reduced pressure to give a brown oil. The crude product was diluted with MeOH (10 mL) at 0 ℃ and AcCl (0.2 mL) was added. After stirred for 2 h, the mixture was concentrated and purified by column chromatography (16:1 petroleum ether:ethyl acetate) to give **S3** (201 mg, 0.85 mmol, 85%) as a clear oil. Data for **S3**: R_f 0.46 (8:1 petroleum ether:ethyl acetate); ^{1}H NMR (400 MHz, $CDCl_3$) δ 3.68 (s, 3 H), 3.30 (t, J = 6.7 Hz, 2 H), 2.49-2.40 (m, 2 H), 2.16 (s, 1 H), 1.80-1.76 (m, 2 H), 1.74-1.66 (m, 2 H), 1.53-1.45 (m, 4 H), 0.94 (t, J = 7.5 Hz, 3 H); ^{13}C NMR (100 MHz, $CDCl_3$) δ 174.0, 88.1, 71.1, 51.7, 51.6, 38.0, 34.6, 32.5, 30.5, 29.6, 24.0, 8.4; IR (thin film) ν_{max} 3293, 2952, 2880, 2097, 1737, 1452, 1437, 1196, 1175; HRMS (ESI) Calcd for $C_{12}H_{20}N_3O_2$ $[M+H]^+$ 238.1556, found: 238.1550.

To a stirred mixture of $Pd(PPh_3)_2Cl_2$ (90 mg, 0.13 mmol, 5 mol%), and CuI (34 mg, 0.18 mmol, 7 mol%) was added a solution of compound **S3** (610 mg, 2.57 mmol, 1 equiv) and **S4** (1.64 g, 5.15 mmol, 2.0 equiv) in a mixture of NEt_3 (6 mL) and THF (6 mL) at -78 ℃ under Argon. The reaction mixture was then degassed by freeze-thaw

cycles before it was stirred at room temperature for 12 h. The reaction mixture was filtered through a pad of Celite. Solvents were removed under reduced pressure and the crude product was purified by column chromatography (16:1 petroleum ether:ethyl acetate) to give compound **15** (892 mg, 2.08 mmol, 81%) as a yellow oil. Data for compound **15**: R_f 0.47 (8:1 petroleum ether:ethyl acetate); ^{1}H NMR (400 MHz, $CDCl_3$) δ 8.07 (d, J = 8.3 Hz, 1 H), 7.33-7.19 (m, 3 H), 6.93 (td, J = 7.6 Hz, 1.1, 1 H), 3.67 (s, 3 H), 3.34 (t, J = 6.5 Hz, 2 H), 2.49 (t, J = 8.0 Hz, 2 H), 1.90 (t, J = 6.4 Hz, 2 H), 1.80-1.70 (m, 2 H), 1.69-1.58 (m, 4 H), 1.51 (s, 9 H), 1.04 (t, J = 7.4 Hz, 3 H); ^{13}C NMR (100 MHz, $CDCl_3$) δ 173.8, 152.3, 139.4, 131.4, 129.1, 122.0, 117.4, 111.4, 100.7, 80.6, 79.1, 51.7, 51.6, 39.1, 34.9, 32.7, 30.9, 29.8, 28.2, 24.2, 8.8; IR (thin film) ν_{max} 3402, 2970, 2937, 2878, 2097, 1735, 1580, 1517, 1449, 1367, 1238, 1157, 755, 591; HRMS (ESI) Calcd for $C_{21}H_{32}N_4O_4Na$ $[M+Na]^+$ 451.2316, found: 451.2316.

Preparation of compound 16

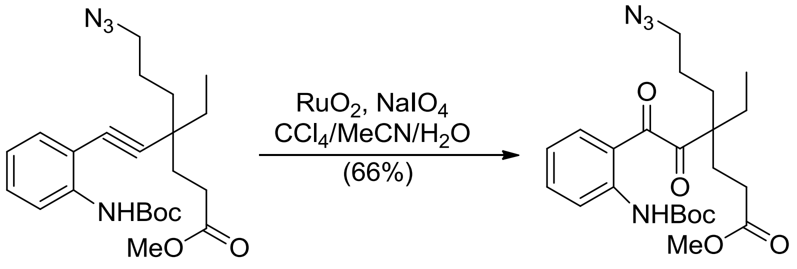

15 **16**

To a solution of compound **15** (730 mg, 1.36 mmol, 1 equiv) in CCl_4 (6 mL) and MeCN (6 mL) was added $NaIO_4$ (732 mg, 3.40 mmol, 2.5 equiv) in H_2O (9 mL). The reaction mixture was stirred vigorously while $RuO_2{\bullet}H_2O$ (9.0 mg, 0.067 mmol, 5 mol%) was added. The reaction mixture was stirred vigorously in air for 2 h before filtered through a pad of silica gel with CH_2Cl_2 as the eluent. The filtrate was washed with an aqueous solution of NaOH (1.0 N, 2×5 mL) and dried over anhydrous Na_2SO_4, and filtered. Solvent was removed under reduced pressure to give a dark color oil. The crude product was purified by column chromatography (8:1 petroleum ether:ethyl acetate) to give compound **16** as a yellow oil (509 mg, 0.89 mmol, 66%). Data for compound **16**: R_f 0.40 (8:1 petroleum ether:ethyl acetate); ^{1}H NMR (400 MHz, $CDCl_3$) δ 10.52 (s, 1 H), 8.55 (d, J = 8.8 Hz, 1 H), 7.57 (t, J = 8.0 Hz, 1 H), 7.40 (d, J = 7.6 Hz, 1 H), 7.01 (t, J = 7.6 Hz, 1 H), 3.67 (s, 3 H), 3.28 (t, J = 6.5 Hz, 2 H), 2.35-2.25 (m, 2 H), 2.10-1.99 (m, 2 H), 1.78-1.68 (m, 4 H), 1.62-1.50 (m, 11 H), 0.88 (t, J = 7.4 Hz, 3 H); ^{13}C NMR (100 MHz, $CDCl_3$) δ 206.7, 197.9, 173.4, 152.6, 143.5, 136.6, 133.1, 121.0, 119.3, 116.7, 81.2, 52.3, 51.8, 51.6, 30.5, 28.7, 28.2, 26.8, 26.3, 23.4, 8.0; IR (thin film) ν_{max} 3402, 2970, 2937, 2878, 2097, 1735, 1580, 1517, 1449, 1367, 1238, 1157, 755, 591; HRMS (ESI) Calcd for $C_{23}H_{33}N_4O_6$ $[M+H]^+$ 461.2395, found: 461.2393.

Preparation of compound 17

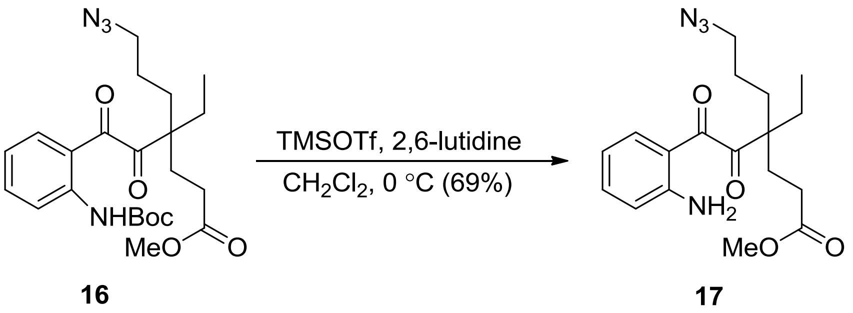

To a solution of compound **16** (35 mg, 0.076 mmol, 1 equiv) and 2,6-lutidine (163 mg, 1.52 mmol, 20 equiv) in CH_2Cl_2 (4 mL) was added TMSOTf (169 mg, 0.76 mmol, 10 equiv) was added dropwise at 0 ℃. The reaction mixture was stirred for 1 h and then diluted with CH_2Cl_2 (10 mL) at 0 ℃, washed with a cold aqueous solution of HCl (1%, 4×5 mL) quickly and dried over anhydrous $MgSO_4$, and filtered. Solvent was removed under reduced pressure to give a brown oil. The crude product was purified by column chromatography (4:1 petroleum ether:ethyl acetate) to give compound **17** as a yellow oil (19 mg, 0.05 mmol, 69%). Data for compound **17**: R_f 0.26 (4:1 petroleum ether:ethyl acetate); ^{1}H NMR (400 MHz, $CDCl_3$) δ 7.34-7.26 (m, 2 H), 6.68 (d, J = 8.0 Hz, 1 H), 6.62 (t, J = 7.6 Hz, 1 H), 6.32 (bs, 2 H), 3.67 (s, 3 H), 3.27 (t, J = 6.7 Hz, 2 H), 2.35-2.28 (m, 2 H), 2.10-2.01 (m, 2 H), 1.78-1.69 (m, 4 H), 1.60-1.52 (m, 2 H), 0.89 (t, J = 7.4 Hz, 3 H); ^{13}C NMR (100 MHz, $CDCl_3$) δ 207.6, 196.4, 173.6, 151.8, 135.8, 132.7, 125.2, 117.4, 116.2, 52.2, 51.7, 30.7, 29.6, 28.9, 28.8, 26.3, 23.5, 8.1; IR (thin film) ν_{max} 3448, 2951, 2879, 1734, 1671, 1581, 1564, 1471, 1296, 1169, 774, 703; HRMS (ESI) Calcd for $C_{18}H_{25}N_4O_4$ $[M+H]^+$ 361.1870, found: 361.1868.[1]

Synthesis of mersicarpine (1)

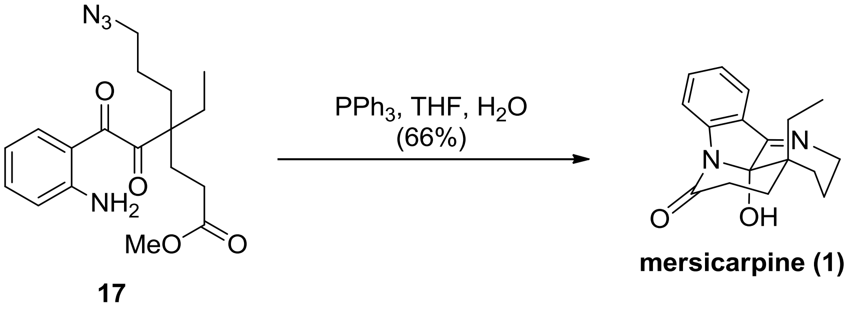

To a solution of compound **17** (10 mg, 0.027 mmol, 1 equiv) in THF (2 mL) and H_2O (0.2 mL) was added PPh_3 (30 mg, 0.111 mmol, 4 equiv). The reaction mixture was stirred at room temperature for 3 days before THF (10 mL) and anhydrous $MgSO_4$ were added. The mixture was filtered and concentrated under reduced pressure. The

crude product was purified by column chromatography (4:1 petroleum ether:ethyl acetate) to give mersicarpine (**1**) (5 mg, 0.018 mmol, 66%) as a light yellow oil. Data for synthetic mersicarpine (**1**): R_f 0.38 (2:1 petroleum ether:ethyl acetate); ^{1}H NMR (400 MHz, $CDCl_3$) δ 8.14 (d, J = 8.4 Hz, 1 H), 7.60 (d, J = 7.6 Hz, 1 H), 7.37 (t, J = 7.8 Hz, 1 H), 7.07 (t, J = 7.4 Hz, 1 H), 3.90-3.84 (m, 2 H), 2.60 (ddd, J = 18.3, 9.5, 3.2 Hz, 1 H), 2.47-2.36 (m, 1 H), 2.05 (d, J = 15.0 Hz, 1 H), 1.97-1.87 (m, 1 H), 1.80-1.72 (m, 1 H), 1.72-1.63 (m, 3 H), 1.39-1.29 (m, 1 H), 1.17-1.08 (m, 1 H), 0.75 (t, J = 7.4 Hz, 3 H); ^{13}C NMR (100 MHz, $CDCl_3$) δ 169.4, 168.4, 146.5, 133.2, 124.4, 124.3, 122.1, 116.8, 93.8, 50.7, 39.3, 34.3, 29.2, 25.4, 23.0, 21.2, 6.8; IR (thin film) ν_{max} 3283, 2955, 2856, 1586, 1485, 1456, 1255, 1081, 969, 838, 757, 674; HRMS (ESI) Calcd for $C_{17}H_{21}N_2O_2$ $[M+H]^+$ 285.1603, found: 285.1602.

Preparation of compound 18

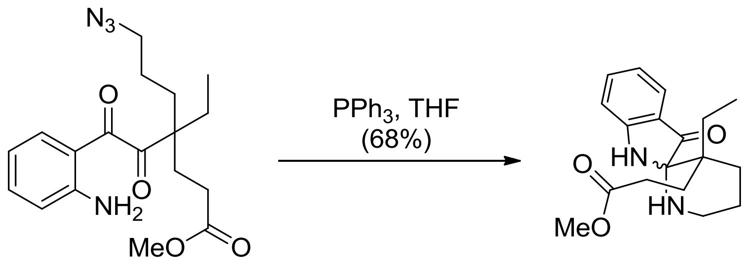

17

18 ***dr*** **= 1:1**

To a solution of compound **17** (10 mg, 0.027 mmol, 1 equiv) in anhydrous THF (2 mL) was added PPh_3 (30 mg, 0.111 mmol, 4 equiv). The solution was stirred at room temperature for 3 days. The mixture was concentrated under reduced pressure and purified by column chromatography (4:1 petroleum ether:ethyl acetate) to give compound **18**, a pair of inseparable diastereomers (1:1, 6 mg, 0.019 mmol, 68%) as a light yellow oil. Data for compound **18**: R_f 0.28 (2:1 petroleum ether:ethyl acetate); ^{1}H NMR (400 MHz, $CDCl_3$) δ 7.56-7.44 (m, 1 H), 7.42-7.34 (m, 1 H), 6.77-6.70 (m, 2 H), 4.64-4.40 (m, 1 H), 3.62 (s, 1.5 H), 3.52 (s, 1.5 H), 3.42-3.25 (m, 1 H), 2.81-2.70 (m, 1 H), 2.34-2.10 (m, 4 H), 2.04-1.93 (m, 1 H), 1.73-1.35 (m, 4 H), 1.33-1.23 (m, 1 H), 0.80 (t, J = 7.3 Hz, 1.5 H), 0.72 (t, J = 7.5 Hz, 1.5 H); ^{13}C NMR (100 MHz, $CDCl_3$) δ 201.5, 201.0, 174.7, 174.4, 159.4, 159.1, 137.1, 125.1, 124.9, 120.2, 120.0, 119.2, 118.9, 112.0, 111.9, 79.97, 79.92, 51.6, 51.4, 39.9, 39.8, 39.7, 39.5, 29.2, 29.1, 28.7, 28.2, 27.7, 27.1, 25.7, 22.4, 21.0, 20.9, 8.5, 7.5; IR (thin film) ν_{max} 3370, 2947, 2878, 1731, 1692, 1617, 1485, 1468, 1437, 1319, 1195, 1076, 753; HRMS (ESI) Calcd for $C_{18}H_{25}N_2O_3$ $[M+H]^+$ 317.1860, found: 317.1858.

Preparation of compound 11

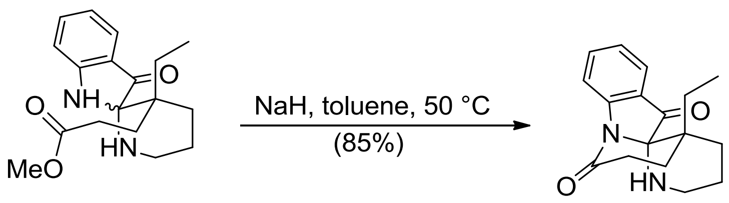

18 ***dr*** **= 1:1** **11**

To a solution of compound **18** (100 mg, 0.32 mmol, 1 equiv) in toluene (5 mL) was added NaH (washed with hexane, 12 mg, 0.50 mmol, 1.5 equiv) in one portion. The suspension was stirred at 50 °C for 12 h before the reaction was quenched with a saturated aqueous solution of NH_4Cl. The mixture was extracted with EtOAc (3×5 mL). The combined solution was dried over anhydrous $MgSO_4$, and filtered. Solvent was removed under reduced pressure. The crude product was purified by column chromatography (4:1 petroleum ether:ethyl acetate) to give compound **11** (77 mg, 0.27 mmol, 85%) as a light yellow oil. Data for compound **11**: R_f 0.45 (2:1 petroleum ether:ethyl acetate); ^{1}H NMR (400 MHz, $CDCl_3$) δ 8.41 (d, J = 8.4 Hz, 1 H), 7.67 (d, J = 7.6 Hz, 1 H), 7.61-7.54 (m, 1 H), 7.17 (t, J = 7.4 Hz, 1 H), 3.22 (dt, J = 12.4, 3.3 Hz, 1 H), 2.91-2.77 (m, 2 H), 2.63-2.43 (m, 2 H), 2.34-2.21 (m, 1 H), 1.86-1.76 (m, 1 H), 1.73-1.51 (m, 4 H), 1.35 (m, 1 H), 1.28-1.18 (m, 1 H), 0.78 (t, J = 7.5 Hz, 3 H); ^{13}C NMR (100 MHz, $CDCl_3$) δ 195.9, 169.6, 151.1, 136.7, 124.3, 124.2, 121.7, 118.2, 80.4, 39.8, 36.6, 29.8, 29.0, 26.4, 23.3, 20.2, 6.8; IR (thin film) ν_{max} 3380, 2965, 2856, 1730, 1693, 1566, 1456, 1255, 1081, 750, 574; HRMS (ESI) Calcd for $C_{17}H_{21}N_2O_2$ $[M+H]^+$ 285.1603, found: 285.1602.

Preparation of compound 19

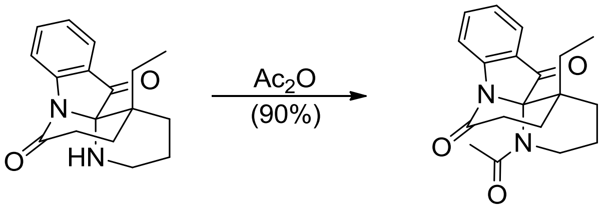

11 **19**

To compound **11** (80 mg, 0.28 mmol, 1 equiv) was added Ac_2O (5 mL). The solution was stirred at room temperature for 12 h before concentrated under reduced pressure. The residue was purified by column chromatography (2:1 petroleum ether:ethyl acetate) to give compound **19** (83 mg, 0.25 mmol, 90%) as a white powder. Data for compound **19**: R_f 0.25 (1:1 petroleum ether:ethyl acetate); ^{1}H NMR (400 MHz, $CDCl_3$) δ 8.42 (d, J = 8.4 Hz, 1 H), 7.67 (d, J = 7.6 Hz, 1 H), 7.62 (t, J = 7.8 Hz, 1 H), 7.18 (t, J = 7.4 Hz, 1 H), 3.86-3.76 (m, 1 H), 3.68-3.60 (m, 1 H), 2.86-2.72 (m, 1 H),

2.54-2.48 (m, 1 H), 2.19-2.08 (m, 1 H), 2.04 (s, 3 H), 2.02-1.90 (m, 4 H), 1.79-1.69 (m, 1 H), 1.18-1.09 (m, 2 H), 0.73 (t, J = 7.5 Hz, 3 H); ^{13}C NMR (100 MHz, $CDCl_3$) δ 196.7, 171.3, 168.9, 152.0, 136.7, 124.0, 123.7, 122.7, 117.0, 81.1, 43.8, 40.6, 30.8, 28.9, 28.1, 26.5, 23.9, 20.2, 7.0; IR (thin film) ν_{max} 2966, 2943, 1729, 1693, 1679, 1452, 1368, 1295, 1008, 767, 756, 574; HRMS (ESI) Calcd for $C_{19}H_{23}N_2O_3$ $[M+H]^+$ 327.1709, found: 327.1709; mp = 200-202 ℃.[2]

Synthesis of leuconodine B (3)

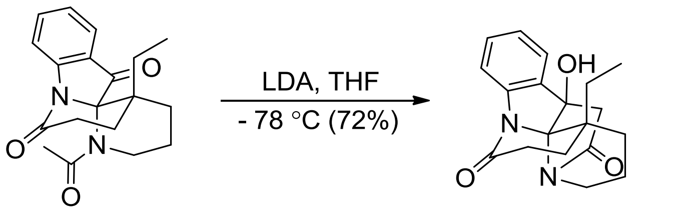

To a solution of compound **19** (10 mg, 0.031 mmol, 1 equiv) in THF (3 mL) was added LDA (2.0 M in THF， 30.0 μL, 0.06 mmol, 2 equiv) at -78 ℃. The reaction mixture was stirred for 2 h before it was quenched by dropwise addition of a saturated aqueous solution of NH_4Cl. The mixture was warmed to room temperature, diluted with water (5 mL), extracted with EtOAc (3×5 mL). The combined solution was dried over anhydrous $MgSO_4$, and filtered. Solvent was removed under reduced pressure. The crude product was purified by column chromatography (4:1 petroleum ether:ethyl acetate) to give leuconodine B (**3**) (7.2 mg, 0.022 mmol, 72%) as a white powder. Data for leuconodine B (**3**): R_f 0.40 (2:1 petroleum ether:ethyl acetate); ^{1}H NMR (400 MHz, $CDCl_3$) δ 7.60 (d, J = 7.6 Hz, 1 H), 7.39 (d, J = 7.2 Hz, 1 H), 7.27 (t, J = 8.0 Hz, 1 H), 7.20 (t, J = 7.4 Hz, 1 H), 4.81 (s, 1 H), 3.89 (d, J = 13.2 Hz, 1 H), 2.97 (d, J = 16.8 Hz, 1 H), 2.87 (d, J = 16.4 Hz, 1 H), 2.52 (t, J = 12.2 Hz, 1 H), 2.29-2.13 (m, 2 H), 1.95-1.87 (m, 1 H), 1.82-1.71 (m, 2 H), 1.61-1.51 (m, 4 H), 1.47-1.41 (m, 1 H), 0.87 (t, J = 7.4, 3 H); ^{13}C NMR (100 MHz, $CDCl_3$) δ 174.2, 169.9, 140.9, 138.3, 129.2, 126.2, 123.3, 121.9, 90.9, 82.1, 42.1, 39.1, 37.0, 28.1, 27.5, 25.9, 22.5, 20.0, 6.9; IR (thin film) ν_{max} 3448, 2949, 2943, 1693, 1646, 1477, 1405, 1363, 1270, 1153, 766, 734; HRMS (ESI) Calcd for $C_{19}H_{23}N_2O_3$ $[M+H]^+$ 327.1709, found: 327.1707; mp = 317-319 ℃.

Preparation of compound 20

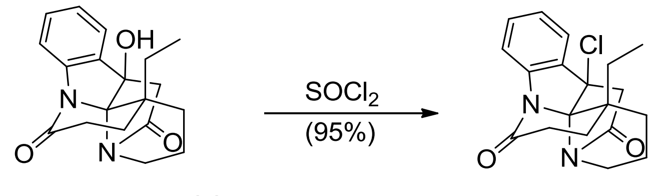

To leuconodine B (**3**) (2 mg, 6.1 μmol, 1 equiv) was added $SOCl_2$ (1.5 mL) at room temperature. The reaction mixture was stirred for 1 h before $SOCl_2$ was removed under reduced pressure. The residue was purified by column chromatography (4:1 petroleum ether:ethyl acetate) to give compound **20** (2 mg, 5.8 μmol , 95%) as a clear oil. Data for compound **20**: R_f 0.41 (2:1 petroleum ether:ethyl acetate); ^{1}H NMR (400 MHz, $CDCl_3$) δ 7.78 (d, J = 8.0 Hz, 1 H), 7.34 (m, 2 H), 7.21 (t, J = 7.6 Hz, 1 H), 3.98 (m, 1 H), 3.27 (d, J = 16.4 Hz, 1 H), 3.20 (d, J = 16.4 Hz, 1 H), 2.89-2.75 (m, 1 H), 2.74-2.50 (m, 3 H), 2.27-2.14 (m, 1 H), 2.07-1.97 (m, 1 H), 1.88-1.77 (m, 1 H), 1.76-1.60 (m, 3 H), 1.57-1.47 (m, 1 H), 0.93 (t, J = 7.0 Hz, 3 H); ^{13}C NMR (100 MHz, $CDCl_3$) δ 172.4, 167.9, 140.0, 136.3, 129.9, 126.2, 122.8, 120.7, 91.8, 69.2, 44.9, 39.9, 37.2, 29.3, 27.8, 25.0, 22.0, 19.8, 6.9; IR (thin film) ν_{max} 2951, 2882, 1704, 1686, 1600, 1477, 1463, 1397, 1352, 1284, 1151, 903, 801, 734; HRMS (ESI) Calcd for $C_{19}H_{22}ClN_2O_2$ $[M+H]^+$ 345.1364, found: 345.1360.

Synthesis of leuconolam (5)

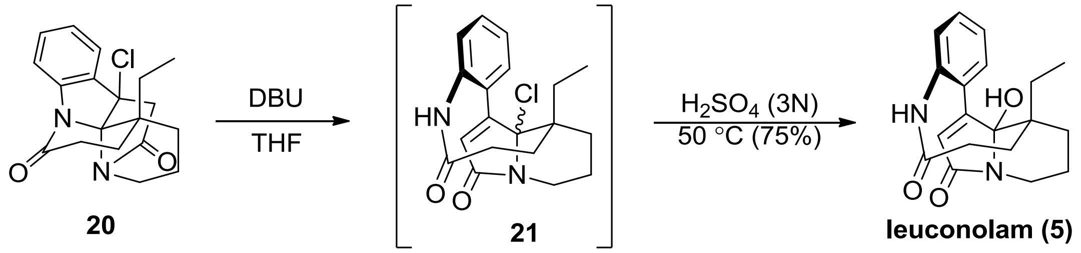

To a solution of compound **20** (12 mg, 0.035 mmol, 1 equiv) in THF (1.5 mL) was added DBU (27 mg, 0.175 mmol, 5.0 equiv). The reaction mixture was stirred at room temperature for 8 h. H_2SO_4 (3N, 2 mL) was added to the mixture, then the mixture was heated to 50 ℃. After stirring for 1 h, the solution was extracted with EtOAc (3×2 mL). The combined solution was dried over anhydrous $MgSO_4$, and filtered. Solvent was removed under reduced pressure. The crude product was purified by column chromatography (1:1 petroleum ether:ethyl acetate) to give leuconolam (**5**) (8.5 mg, 0.026 mmol, 75%) as a white solid. Data for leuconolam (**5**): R_f 0.22 (9:1 $CHCl_3$:MeOH); ^{1}H NMR (400 MHz, $CDCl_3$:CD_3OD = 3:1(v:v)) δ 7.81 (d, J = 8.0 Hz, 1 H), 7.40 (t, J = 7.6 Hz, 1 H), 7.32 (t, J = 7.6 Hz, 1 H), 7.22 (d, J = 7.6 Hz, 1 H), 5.92 (s, 1 H), 4.01-3.97 (m, 1 H), 2.95 (dt, J = 12.8, 4.8 Hz, 1 H), 2.16 (dd, J = 14.7, 7.4

Hz, 1 H), 1.98 (t, J = 12.9 Hz, 1 H), 1.83-1.72 (m, 1 H), 1.66-1.40 (m, 6 H), 1.30-1.20 (m, 1 H), 0.55 (t, J = 7.5 Hz, 3 H); ^{13}C NMR (100 MHz, $CDCl_3$:CD_3OD = 3:1(v:v)) δ 178.6, 166.8, 156.3, 135.0, 133.0, 129.6, 128.9, 127.7, 126.6, 126.1, 93.5, 45.0, 35.1, 32.0, 27.5, 25.3, 24.1, 19.7, 6.7; IR (thin film) ν_{max} 3737, 3067, 2963, 2945, 2870, 1677, 1648, 1458, 1438, 1385, 1261, 1086, 1027, 805; HRMS (ESI) Calcd for $C_{19}H_{23}N_2O_2$ $[M+H]^+$ 327.1703, found: 327.1701; mp = 198-200 ℃.

Synthesis of melodinine E (4)

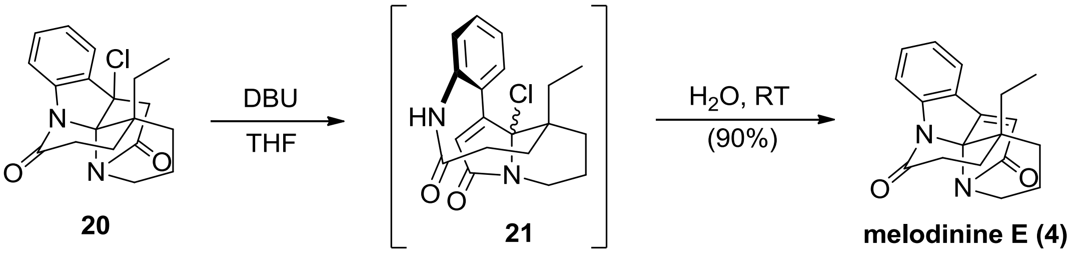

To a solution of compound **20** (12 mg, 0.035 mmol, 1 equiv)in THF (1.5 mL) was added DBU (27 mg, 0.175 mmol, 5.0 equiv). The reaction mixture was stirred at room temperature for 8 h. Water (2 mL) was added to the mixture, and the mixture was extracted with EtOAc (3×2 mL). The combined solution was dried over anhydrous $MgSO_4$, and filtered. Solvent was removed under reduced pressure. The crude product was purified by column chromatography (4:1 petroleum ether:ethyl acetate) to give melodinine E (**4**) (9.7 mg, 0.031 mmol, 90%) as a clear oil. Data for melodinine E (**4**): R_f 0.40 (2:1 petroleum ether:ethyl acetate); ^{1}H NMR (400 MHz, $CDCl_3$) δ 8.16 (d, J = 8.4 Hz, 1 H), 7.45 (d, J = 7.2 Hz, 1 H), 7.33 (t, J = 7.4 Hz, 1 H), 7.12 (t, J = 7.4 Hz, 1 H), 6.21 (s, 1 H), 4.47 (ddd, J = 15.2, 11.7, 3.7, 1 H), 3.26-3.16 (m, 1 H), 3.09 (dt, J = 15.7, 6.2 Hz, 1 H), 2.64-2.58 (m, 1 H), 2.13-2.00 (m, 2 H), 1.83-1.63 (m, 3 H), 1.49-1.41 (m, 1 H), 1.40-1.30 (m, 1 H), 1.10 (dt, J = 13.5, 6.8 Hz, 1 H), 0.76 (t, J = 7.3 Hz, 3 H); ^{13}C NMR (100 MHz, $CDCl_3$) δ 176.1, 173.5, 164.3, 148.7, 131.6, 124.3, 123.5, 121.6, 118.2, 116.0, 93.6, 44.6, 37.0, 34.2, 33.1, 30.5, 26.1, 16.8, 8.3; IR (thin film) ν_{max} 2963, 2880, 1689, 1648, 1597, 1456, 1371, 1356, 1261, 1095, 1021, 801; HRMS (ESI) Calcd for $C_{19}H_{21}N_2O_2$ $[M+H]^+$ 309.1603, found: 309.1601.

Synthesis of leuconoxine (2)

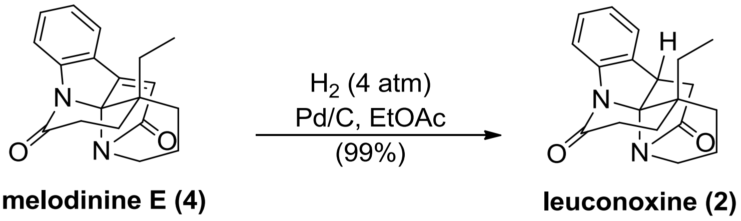

To a solution of melodinine E (**4**) (2.0 mg, μmol) in EtOAc (5 mL) was added Pd/C

(0.2 mg, 10% w/w). The reaction mixture was purged with H_2 and stirred under a pressure of H_2 (4 atm) at room temperature for 8 h. The reaction mixture was filtered through a pad of Celite, and washed with EtOAc (3×5 mL). The filtrate was concentrated under reduced pressure and purified by column chromatography (4:1 petroleum ether:ethyl acetate) to give leuconoxine (**2**) (2.0 mg, 6.5 μmol, 99%) as a white powder. Data for leuconoxine (**2**): R_f 0.30 (1:1 petroleum ether:ethyl acetate); ^{1}H NMR (400 MHz, $CDCl_3$) δ 7.77 (d, J = 8.0 Hz, 1 H), 7.26-7.22 (m, 1 H), 7.19-7.12 (m, 2 H), 3.95 (d, J = 13.2 Hz, 1 H), 3.82 (d, J = 7.2 Hz, 1 H), 2.92-2.66 (m, 4 H), 2.54-2.45 (m, 1 H), 2.02-1.92 (m, 1 H), 1.90-1.76 (m, 2 H), 1.70-1.54 (m, 4 H), 1.43-1.32 (m, 1 H), 0.92 (t, J = 7.2 Hz, 3 H); ^{13}C NMR (100 MHz, $CDCl_3$) δ 172.9, 170.8, 142.0, 135.1, 128.0, 125.5, 123.9, 120.1, 92.6, 41.9, 38.1, 37.6, 36.8, 29.4, 26.9, 26.6, 26.2, 20.1, 7.3; IR (thin film) ν_{max} 2947, 2867, 1677, 1476, 1457, 1397, 1361, 1143, 1086, 888, 761; HRMS (ESI) Calcd for $C_{19}H_{23}N_2O_2$ $[M+H]^+$ 311.1760, found: 311.1754; mp = 188-190 ℃.

Preparation of (-)-12

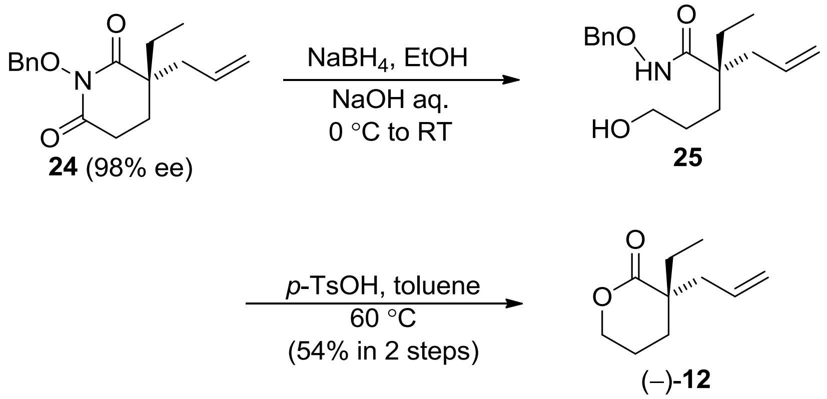

To a solution of **24** (143 mg, 0.498 mmol) in ethanol (10 mL) was added $NaBH_4$ (188 mg, 4.98 mmol) portionwise at 0 ℃. After the starting material was shown to be consumed by TLC analysis, 2 M aqueous NaOH (0.30 mL) was added to the reaction mixtureat 0 ℃. The resulting mixture was slowly warmed up to room temperature and stirred overnight. 1M aqueous HCl, saturated Rochelle's salt aqueous solution and CH_2Cl_2 were added to the reaction mixture. The mixture was stirred for 2 h and the phases were separated. The aqueous phase was extracted with CH_2Cl_2 twice. The combined organic phases were dried over Na_2SO_4, filtered and concentrated under reduced pressure. The crude product was used for the next reaction without further purification.

To a solution of **25** in toluene (5.0 mL) was added *p*-toluenesulfonic acid monohydrate (114 mg, 0.598 mmol) and the reaction mixture was heated at 60 ℃ for 3 h. The reaction mixture was cooled to room temperature and purified by flash column chromatography (6:1 petroleum ether:ethyl acetate) to afford (–)-**12** (45.0 mg, 0.267 mmol, 54% in 2 steps) as a colorless oil. Data for (–)-**12**: R_f = 0.35 (6:1

petroleum ether:ethyl acetate); ^{1}H NMR (500 MHz, $CDCl_3$) δ 5.75 (m, 1 H), 5.14–5.07 (m, 2 H), 4.29 (m, 2 H), 2.53 (m, 1 H), 2.19 (m, 1 H), 1.88–1.72 (m, 5 H), 1.55 (m, 1 H), 0.92 (t, J = 7.5 Hz, 3 H); ^{13}C NMR (126 MHz, $CDCl_3$) δ 175.4, 133.8, 118.9, 70.2, 46.5, 43.8, 32.4, 28.9, 21.4, 8.9; IR (Neat Film,NaCl) 2966, 1724, 1453, 1397, 1249, 1142, 1075, 917; HRMS (ESI+) *m/z* calc'd for $C_{10}H_{17}O_2$ $[M+H]^+$: 169.1223, found 169.1218; $[\alpha]_D^{25}$ = –8.0 (c = 0.97, $CHCl_3$).

Table 1: Chemical shifts of ^{13}C NMR data for natural, synthetic mersicarpine (1) reported previously and our synthetic mersicarpine (1).

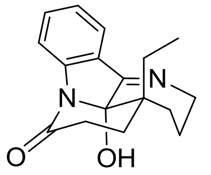

mersicarpine (1)

Natural mersicarpine (Prof. Kam, T.-S.) [3] (100 MHz, $CDCl_3$)	Synthetic mersicarpine (Prof. Kerr, M. A.)[4] (100 MHz, $CDCl_3$)	Synthetic mersicarpine (Prof. Liang, G. X.) (100 MHz, $CDCl_3$)
169.6	169.5	169.4
168.9	168.8	168.4
146.5	146.5	146.5
133.2	133.2	133.2
124.4	124.3	124.4
124.2	124.3	124.1
122.2	122.2	122.1
116.7	116.7	116.8
93.8	93.8	93.8
50.5	50.5	50.7
39.3	39.3	39.3
34.3	34.3	34.3
29.1	29.1	29.2
25.4	25.4	25.4
22.9	22.9	23.0
21.1	21.1	21.2
6.8	6.8	6.8

Table 2: Chemical shifts of ^{13}C NMR data for natural, synthetic leuconodine B (3) reported previously and our synthetic leuconodine B (3).

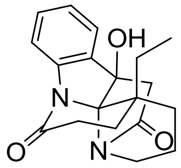

leuconodine B (3)

Natural leuconodine B (Prof. Kam, T.-S.) [5] (100 MHz, $CDCl_3$)	Natural scholarisine G (Prof. Luo, X.-D.)[6] (100 MHz, $CDCl_3$)	Synthetic leuconodine B (Prof. Zhu, J.-P.) [2] (100 MHz, $CDCl_3$)	Synthetic leuconodine B (Prof. Liang, G. X.) (100 MHz, $CDCl_3$)
173.6	173.2	173.6	174.2
170.0	169.9	170.0	169.9
141.3	141.3	141.5	140.9
137.4	136.9	137.3	138.3
129.4	129.9	129.9	129.2
125.6	125.8	125.9	126.2
122.9	122.7	123.0	123.3
120.7	120.8	120.9	121.9
90.4	90.5	90.7	90.9
81.9	82.0	82.1	82.1
42.2	42.1	42.3	42.1
39.1	39.1	39.3	39.1
36.8	36.8	37.0	37.0
29.5	29.5	29.6	28.1
27.2	27.2	27.4	27.5
25.5	25.6	25.7	25.9
22.4	22.5	22.7	22.5
20.0	20.0	20.2	20.0
6.9	6.9	7.1	6.9

Table 3: Chemical shifts of ^{13}C NMR data for natural, synthetic leuconolam (5) reported previously and our synthetic leuconolam (5).

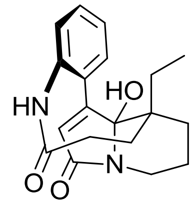

leuconolam (5)

Natural (–)-leuconolam (Prof. Goh.S.H.)[7] (100 MHz, $CDCl_3$)	Synthetic (±)-leuconolam (Prof. Zhu, J.-P.)[2] (100 MHz, $CDCl_3/CD_3OD$ = 3/1)	Synthetic (±)-leuconolam (Prof. Liang, G. X.) (100 MHz, $CDCl_3/CD_3OD$ = 3/1)
177.8	179.3	178.6
166.5	167.5	166.7
155.6	157.0	156.3
135.0	135.7	135.0
133.1	133.7	133.0
129.4	130.3	129.6
129.3	129.6	128.9
128.1	128.4	127.7
126.6	127.3	126.6
126.3	126.9	126.1
93.6	94.2	93.5
44.9	45.7	45.0
35.3	35.8	35.1
32.1	32.7	32.0
27.3	28.3	27.5
25.4	26.0	25.3
24.5	24.8	24.0
19.7	20.4	19.7
6.9	7.5	6.7

Table 4: Chemical shifts of ^{13}C NMR data for natural, synthetic melodinine E (4) reported previously and our synthetic melodinine E (4).

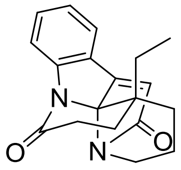

melodinine E (4)

Natural melodinine E (Prof. Luo, X.-D.)[8] (100 MHz, $CDCl_3$)	Synthetic melodinine E (Prof. Zhu, J.-P.) [2] (100 MHz, $CDCl_3$)	Synthetic melodinine E (Prof. Liang, G. X.) (100 MHz, $CDCl_3$)
176.0	176.2	176.1
173.5	173.6	173.5
164.2	164.4	164.3
148.5	148.8	148.7
131.5	131.7	131.6
124.3	124.5	124.3
123.4	123.6	123.5
121.5	121.7	121.6
118.1	118.3	118.2
115.8	116.0	116.0
93.5	93.8	93.6
44.5	44.7	44.6
36.9	37.1	37.0
34.1	34.3	34.2
33.1	33.2	33.1
30.4	30.5	30.5
26.0	26.2	26.1
16.7	16.9	16.8
8.2	8.4	8.3

Table 5: Chemical shifts of ^{13}C NMR data for natural, synthetic leuconoxine (2) reported previously and our synthetic leuconoxine (2).

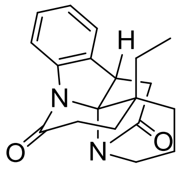

leuconoxine (2)

Natural leuconoxine (Prof. Abe, F.)[9] (100 MHz, $CDCl_3$)	Synthetic leuconoxine (Prof. Baudoin, O.)[10] (100 MHz, $CDCl_3$)	Synthetic leuconoxine (Prof. Zhu, J.-P.) [2] (100 MHz, $CDCl_3$)	Synthetic leuconoxine (Prof. Liang, G. X.) (100 MHz, $CDCl_3$)
172.8	173.0	173.1	172.9
170.7	171.0	171.0	170.8
142.0	141.6	142.1	142.0
135.1	135.3	135.2	135.1
127.9	128.2	128.1	128.0
125.5	125.7	125.7	125.5
123.8	124.0	124.0	123.9
120.1	120.3	120.2	120.1
92.5	92.2	92.7	92.6
41.9	42.1	42.1	41.9
38.1	38.3	38.2	38.1
37.5	37.8	37.7	37.6
36.8	37.0	36.9	36.8
29.4	29.6	29.5	29.4
27.0	27.1	27.0	26.9
26.7	26.9	26.7	26.6
27.3	26.4	26.4	26.2
20.1	20.3	20.2	20.1
7.3	7.5	7.4	7.3

Reference and Note:

[1] Compound **17** is unstable while being kept at room temperature for a long time. It is also sensitive to acid, base and heat. Therefore, it could not be isolated with high purity.

[2] Xu, Z.; Wang, Q.; Zhu, J. *J. Am. Chem. Soc.* **2013**, *135*, 19127–19130.

[3] Kam, T.-S.; Subramaniam, G.; Lim, K.-H.; Choo, Y.-M. *Tetrahedron Lett.* **2004**, *45*, 5995–5998.

[4] Magolan, J.; Carson, C. A.; Kerr, M. A. *Org. Lett.* **2008**, *10*, 1437–1440.

[5] Gan, C.-Y.; Low, Y.-Y.; Thomas, N. F.; Kam, T.-S. *J. Nat. Prod*. **2013**, *76*, 957–964.

[6] Feng, T.; Cai, X.-H.; Zhao, P.-J.; Du, Z.-Z.; Li, W.-Q.; Luo, X.-D. *Planta Med*. **2009**, *75*, 1537–1541.

[7] S.H. GOH.; Razak Mohd Ali, A.; Wong , W.H.**.** *Tetrahedron*, **1989**, *45*, 7899–7920

[8] Feng, T.; Cai, X.-H.; Liu, Y.-P.; Li, Y.; Wang, Y.-Y.; Luo, X.-D. *J. Nat. Prod*. **2010,** *73*, 22–26.

[9] Abe, F.; Yamaguchi, T. *Phytochemistry* **1994**, *35*, 169–171.

[10] De′cor, A.; Bellocq, D.; Thoison, O.; Lekieffre, N.; Chiaroni, A.; Ouazzani, J.; Cresteil, T.; Gue′ritte, F.; Baudoin, O. *Bioorg. Med. Chem.* **2006**, *14*, 1558–1564.

^{1}H NMR and ^{13}C NMR Spectra

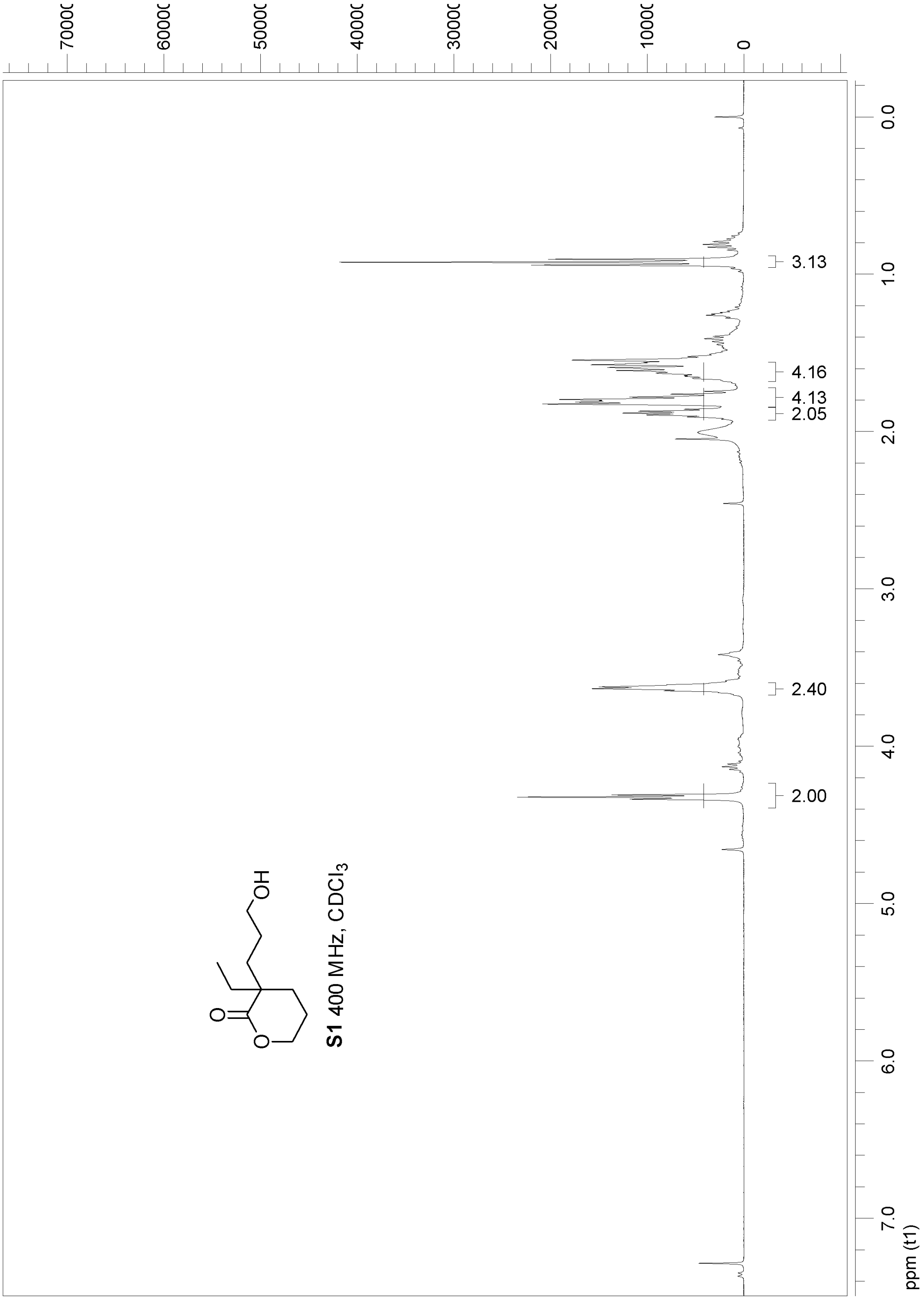

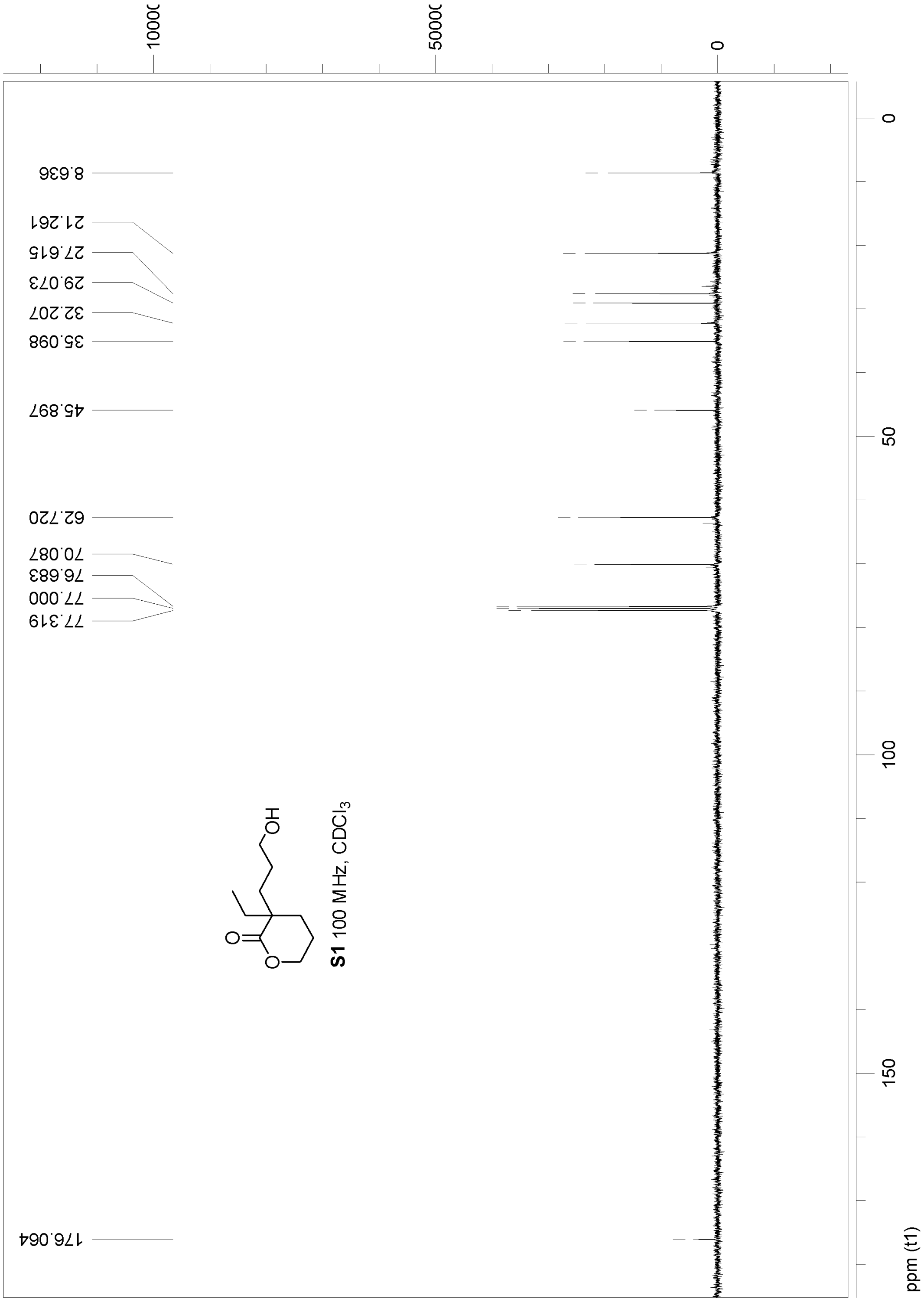

S1 100 MHz, CDCl3
OH
O
O
8.636
21.261
27.615
29.073
32.207
35.098
45.897
62.720
70.087
76.683
77.000
77.319
176.064
10000
50000
0
0
50
100
150
ppm (t1)

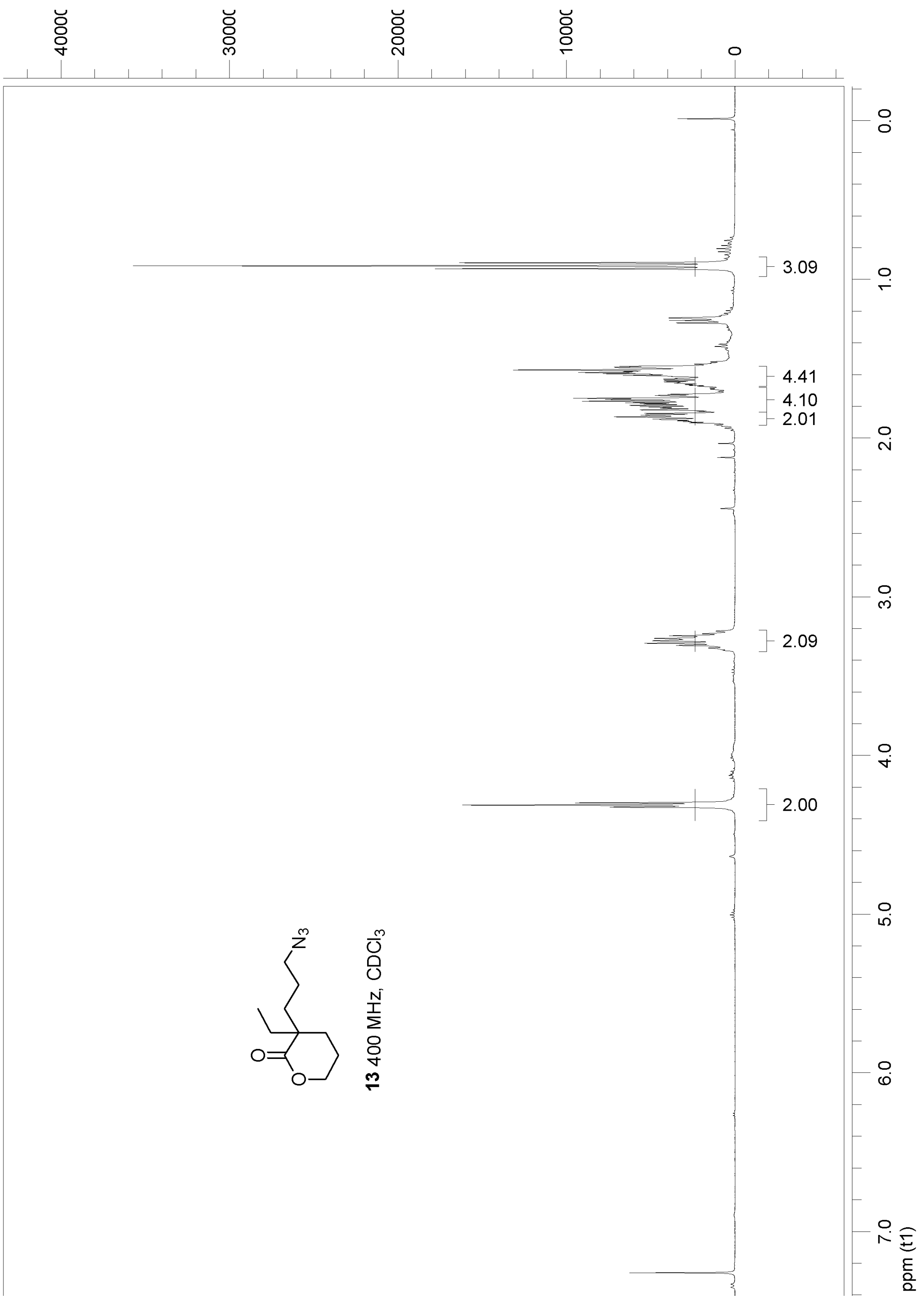
40000
30000
20000
10000
0
3.09
4.41
4.10
2.01
2.09
2.00
0.0
1.0
2.0
3.0
4.0
5.0
6.0
7.0
ppm (t1)
O
O
N3
13 400 MHz, CDCl3

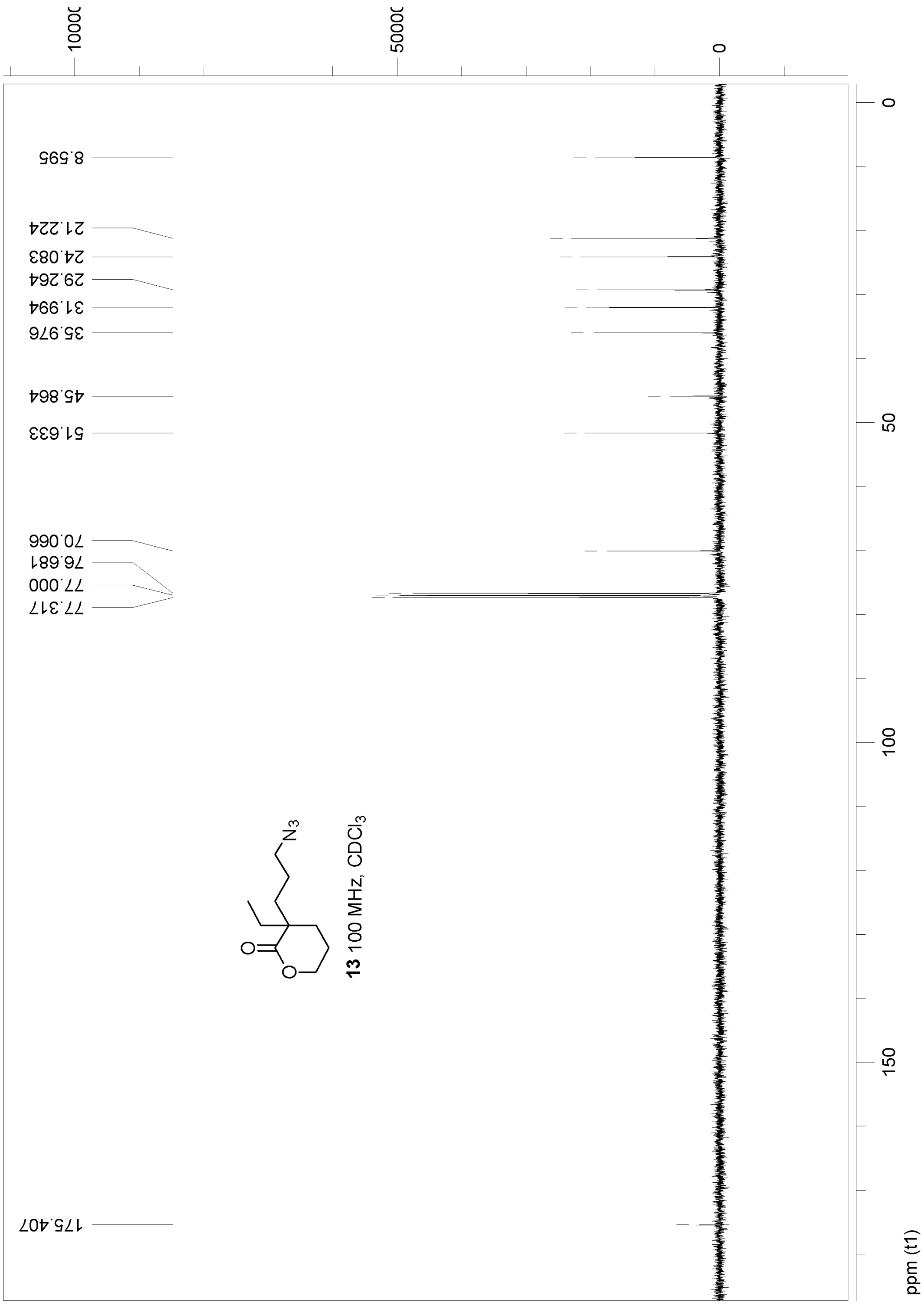

100000
50000
0
8.595
21.224
24.083
29.264
31.994
35.976
45.864
51.633
70.066
76.681
77.000
77.317
175.407
O
O
N_3
13 100 MHz, $CDCl_3$
0
50
100
150
ppm (t1)

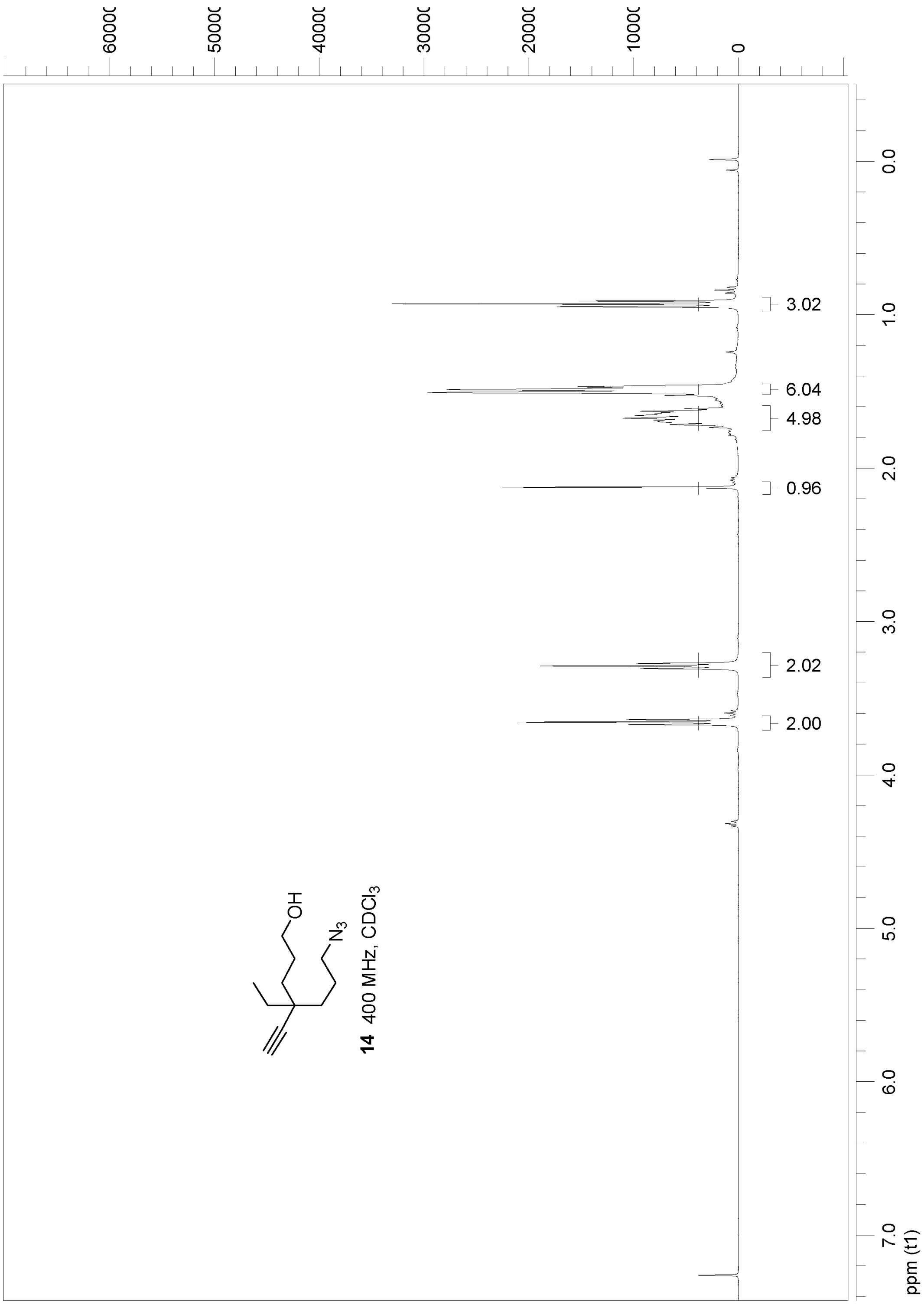
60000
50000
40000
30000
20000
10000
0
3.02
6.04
4.98
0.96
2.02
2.00
OH
N3
14 400 MHz, CDCl3
0.0
1.0
2.0
3.0
4.0
5.0
6.0
7.0
ppm (t1)

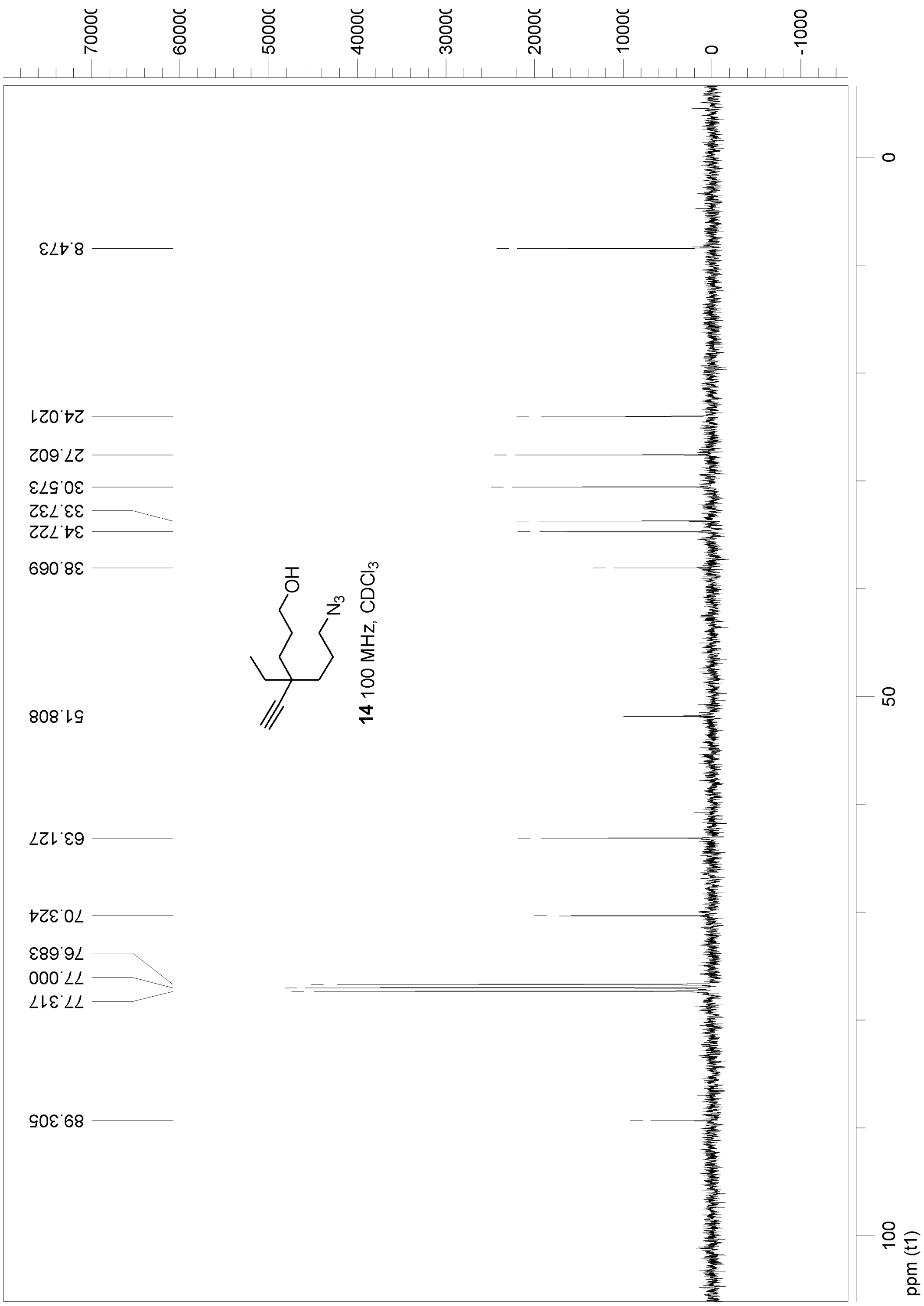
8.473
24.021
27.602
30.573
33.732
34.722
38.069
51.808
63.127
70.324
76.683
77.000
77.317
89.305
OH
N3
14 100 MHz, CDCl3
0
50
100
ppm (t1)

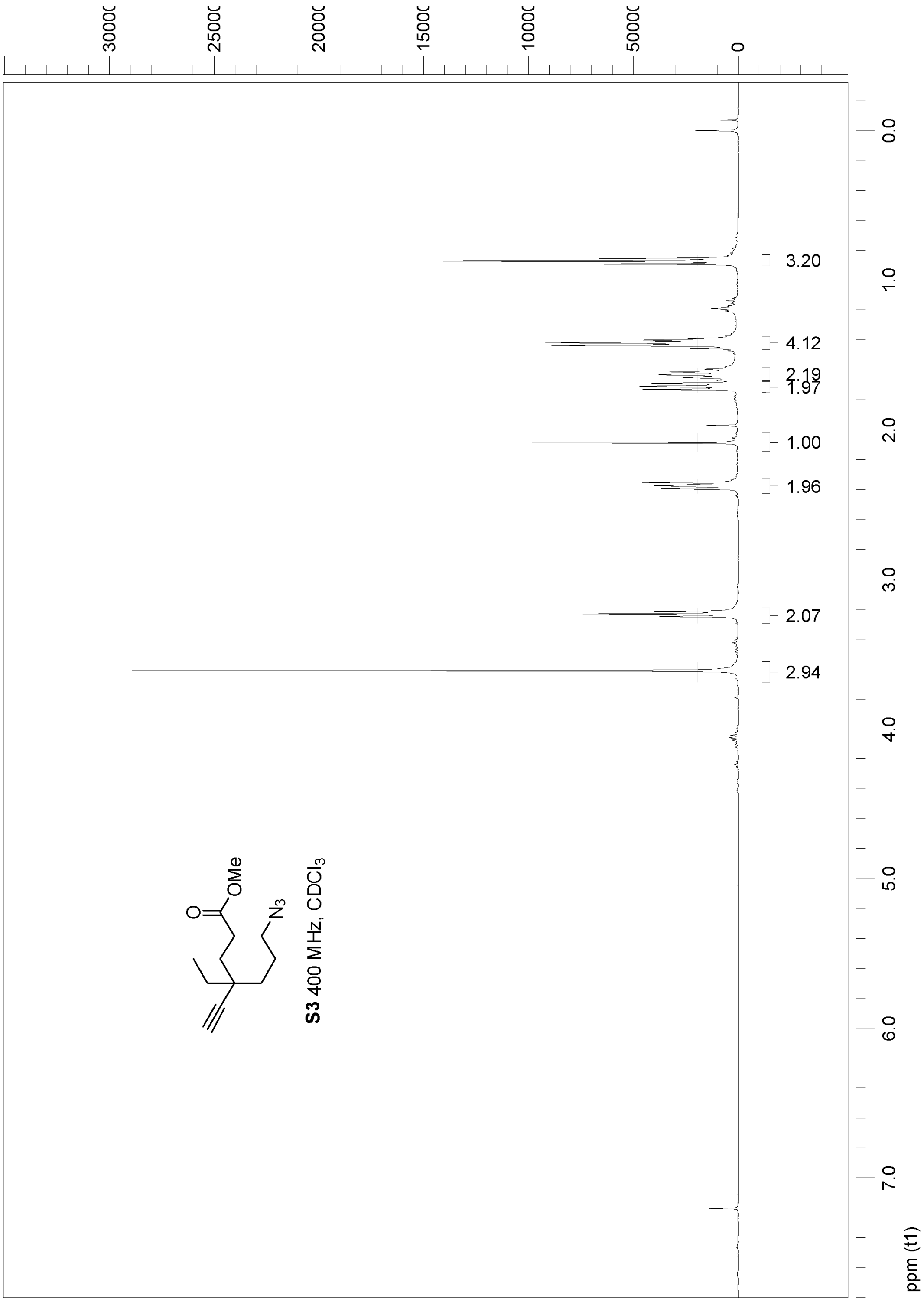

30000
25000
20000
15000
10000
50000
0
3.20
4.12
2.19
1.97
1.00
1.96
2.07
2.94
0.0
1.0
2.0
3.0
4.0
5.0
6.0
7.0
ppm (t1)
O
OMe
N_3
S3 400 MHz, $CDCl_3$

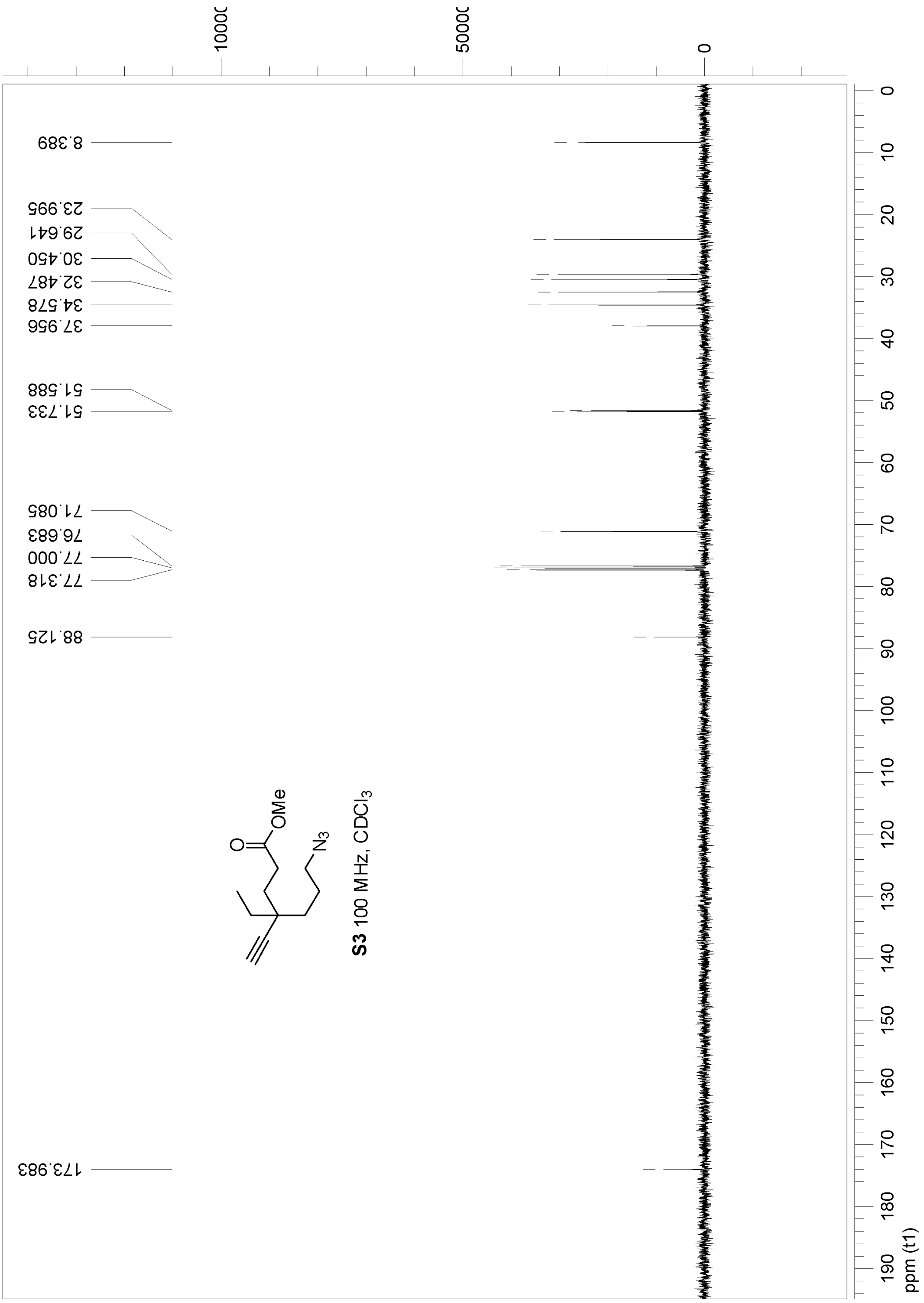

8.389
23.995
29.641
30.450
32.487
34.578
37.956
51.588
51.733
71.085
76.683
77.000
77.318
88.125
173.983
OMe
O
N3
S3 100 MHz, CDCl3
ppm (t1)

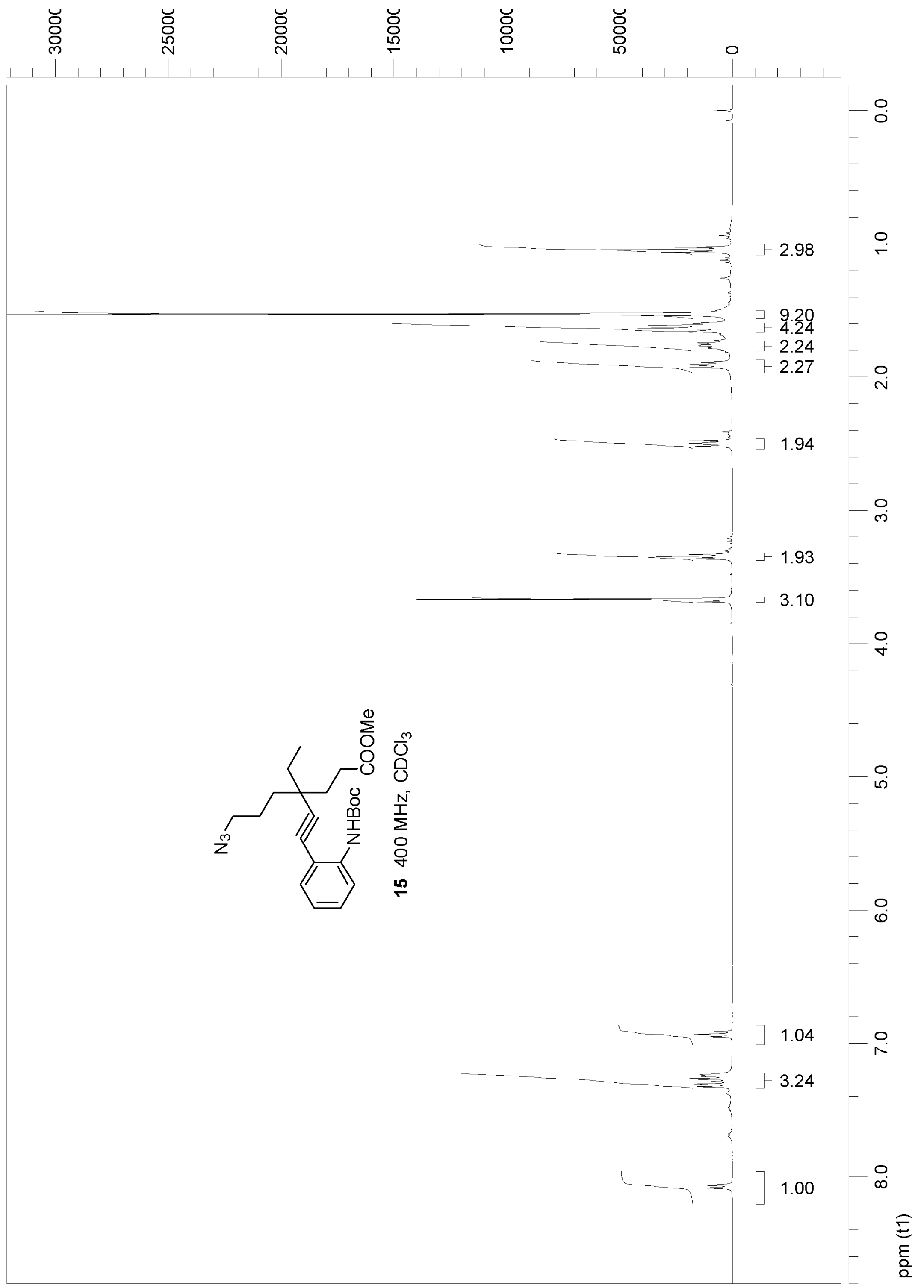

N3
NHBoc
COOMe
15 400 MHz, CDCl3
2.98
9.20
4.24
2.24
2.27
1.94
1.93
3.10
1.04
3.24
1.00
ppm (t1)

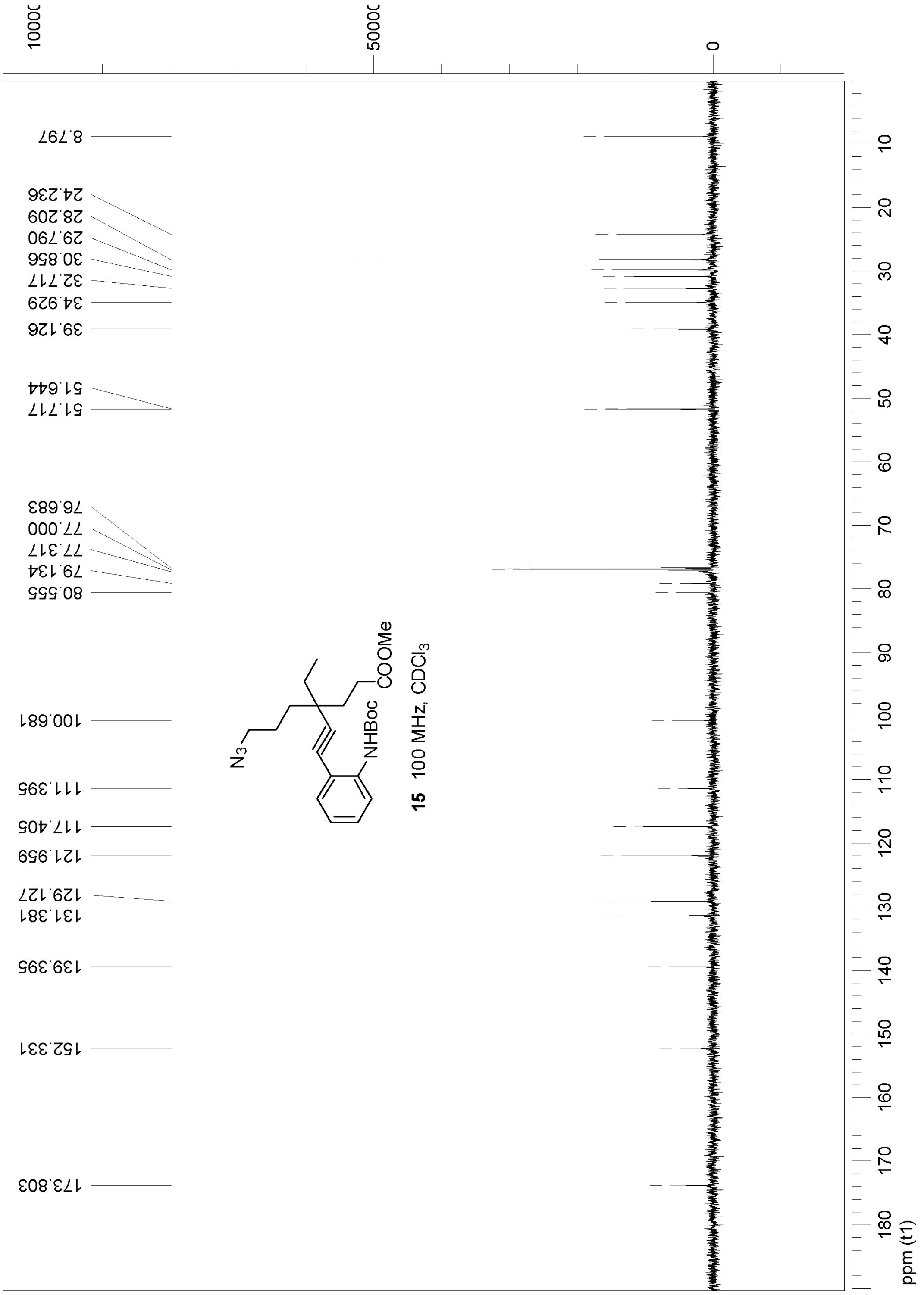
8.797
24.236
28.209
29.790
30.856
32.717
34.929
39.126
51.644
51.717
76.683
77.000
77.317
79.134
80.555
100.681
111.395
117.405
121.959
129.127
131.381
139.395
152.331
173.803
N3
NHBoc
COOMe
15 100 MHz, CDCl3
ppm (t1)

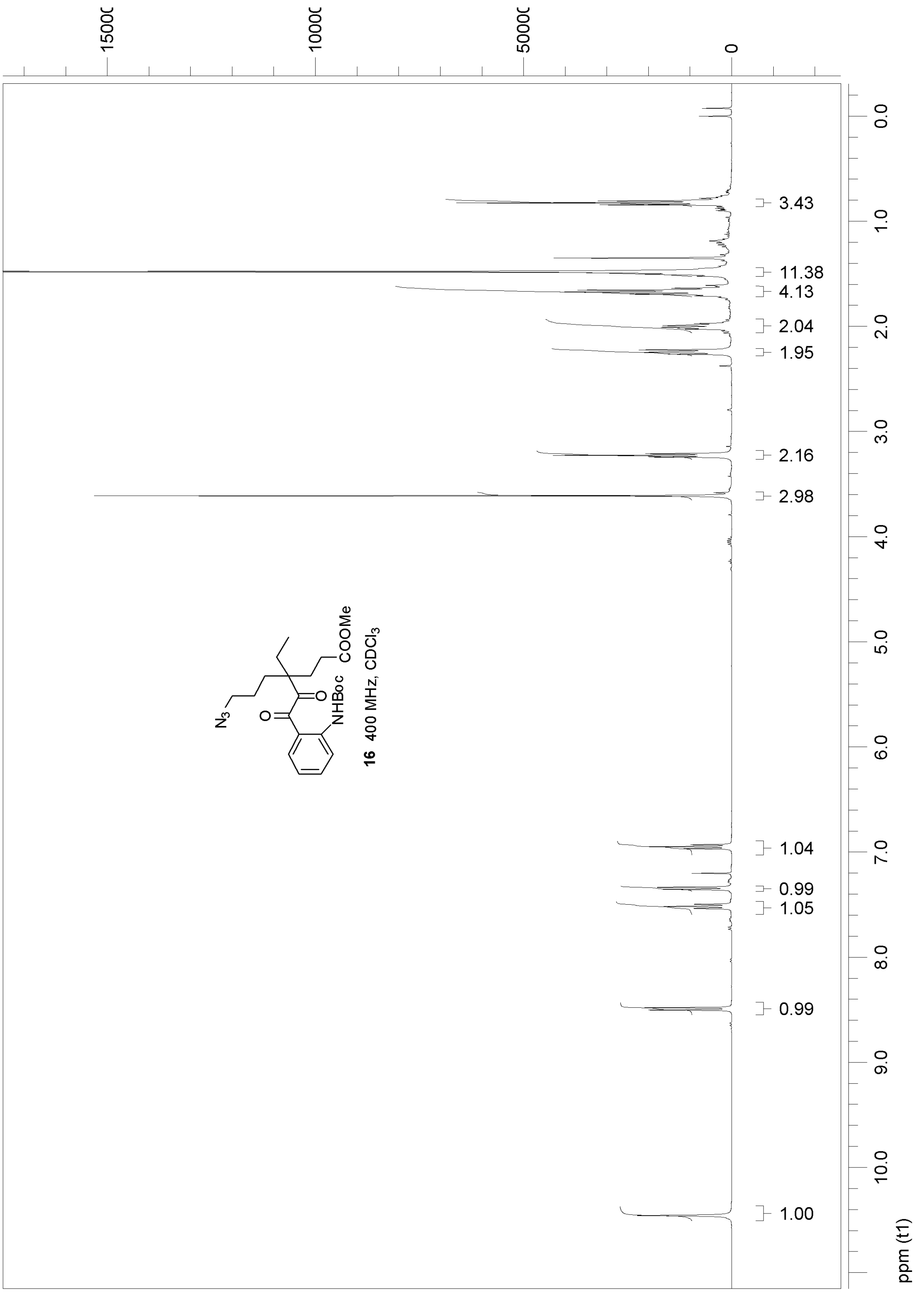
15000
10000
5000
0
3.43
11.38
4.13
2.04
1.95
2.16
2.98
1.04
0.99
1.05
0.99
1.00
ppm (t1)
N3
O
O
NHBoc
COOMe
16 400 MHz, CDCl3

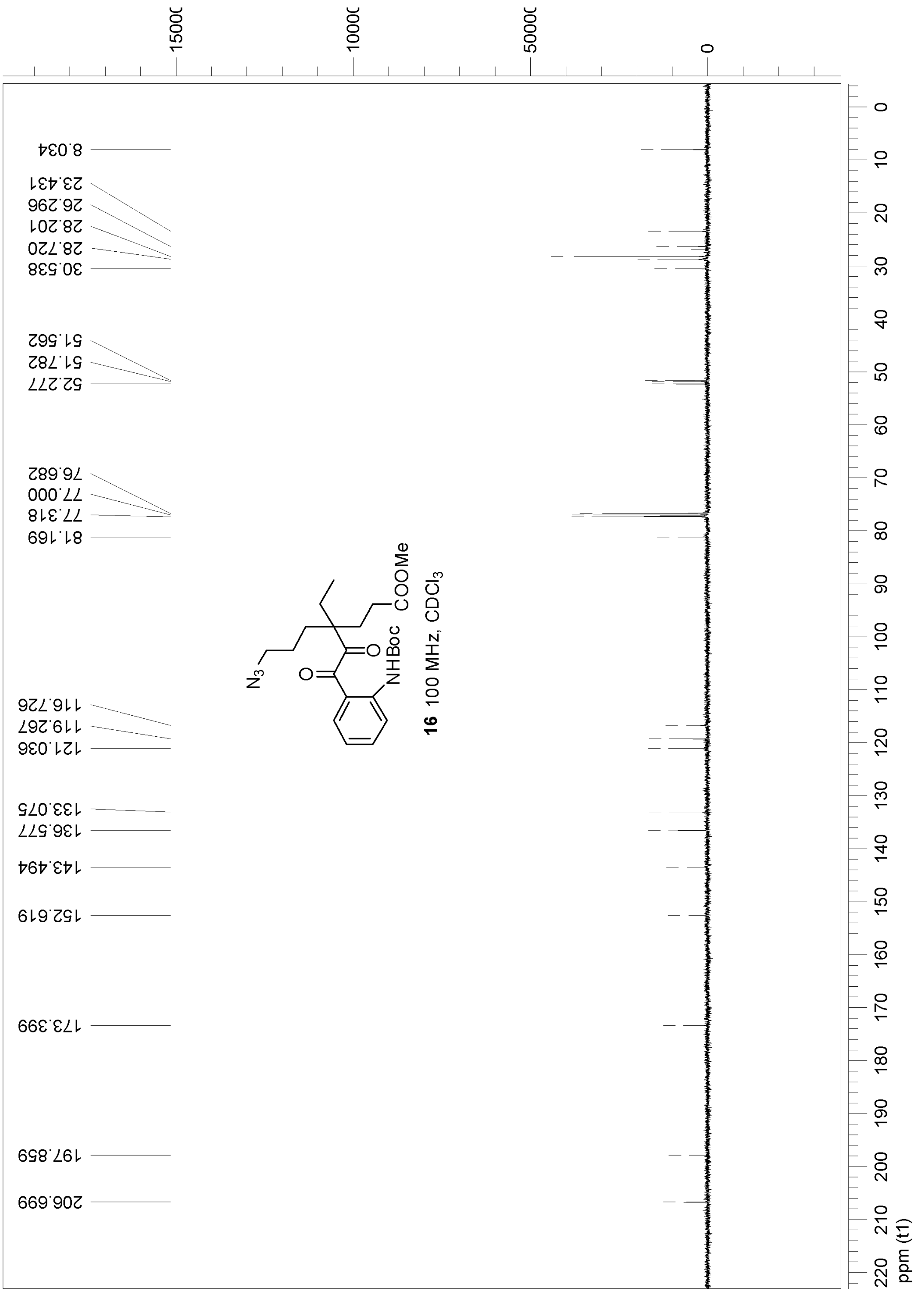

16 100 MHz, $CDCl_3$

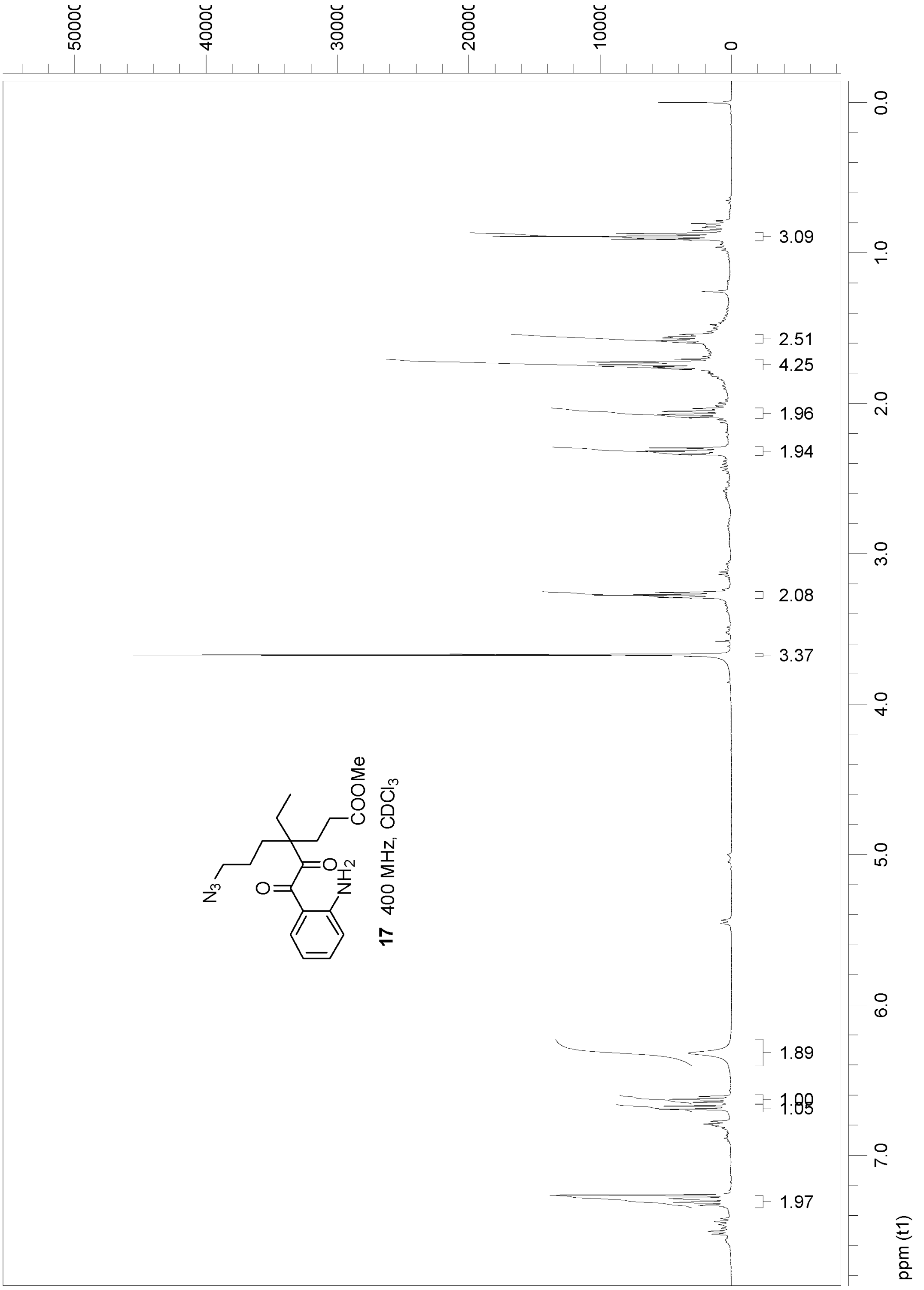

N3
O
O
NH2
COOMe
17 400 MHz, CDCl3
50000
40000
30000
20000
10000
0
3.09
2.51
4.25
1.96
1.94
2.08
3.37
1.89
1.00
1.05
1.97
0.0
1.0
2.0
3.0
4.0
5.0
6.0
7.0
ppm (t1)

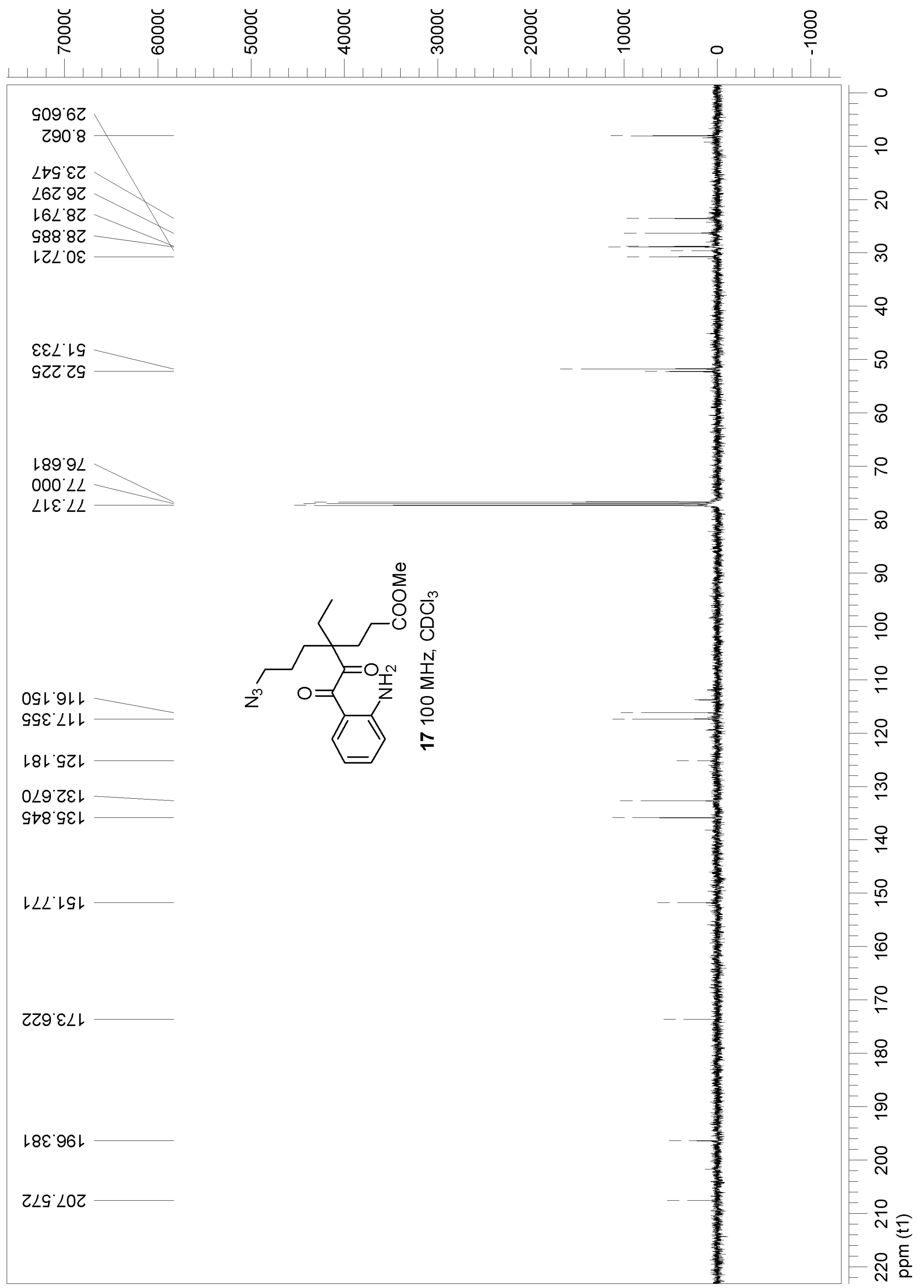
29.605
8.062
23.547
26.297
28.791
28.885
30.721
51.733
52.225
76.681
77.000
77.317
116.150
117.355
125.181
132.670
135.845
151.771
173.622
196.381
207.572
COOMe
N_3
NH_2
17 100 MHz, $CDCl_3$
ppm (t1)

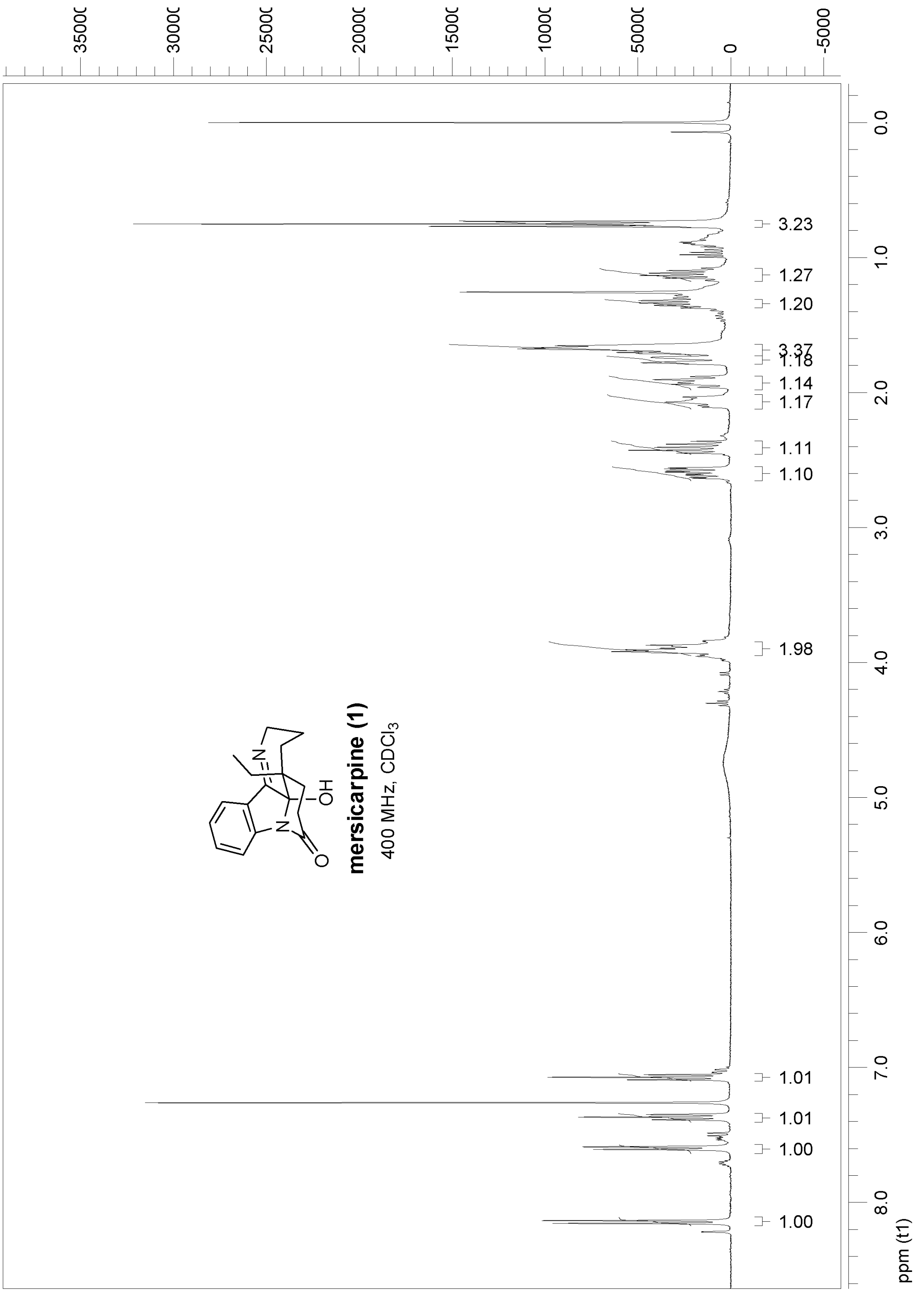
mersicarpine (1)
400 MHz, CDCl3
ppm (t1)

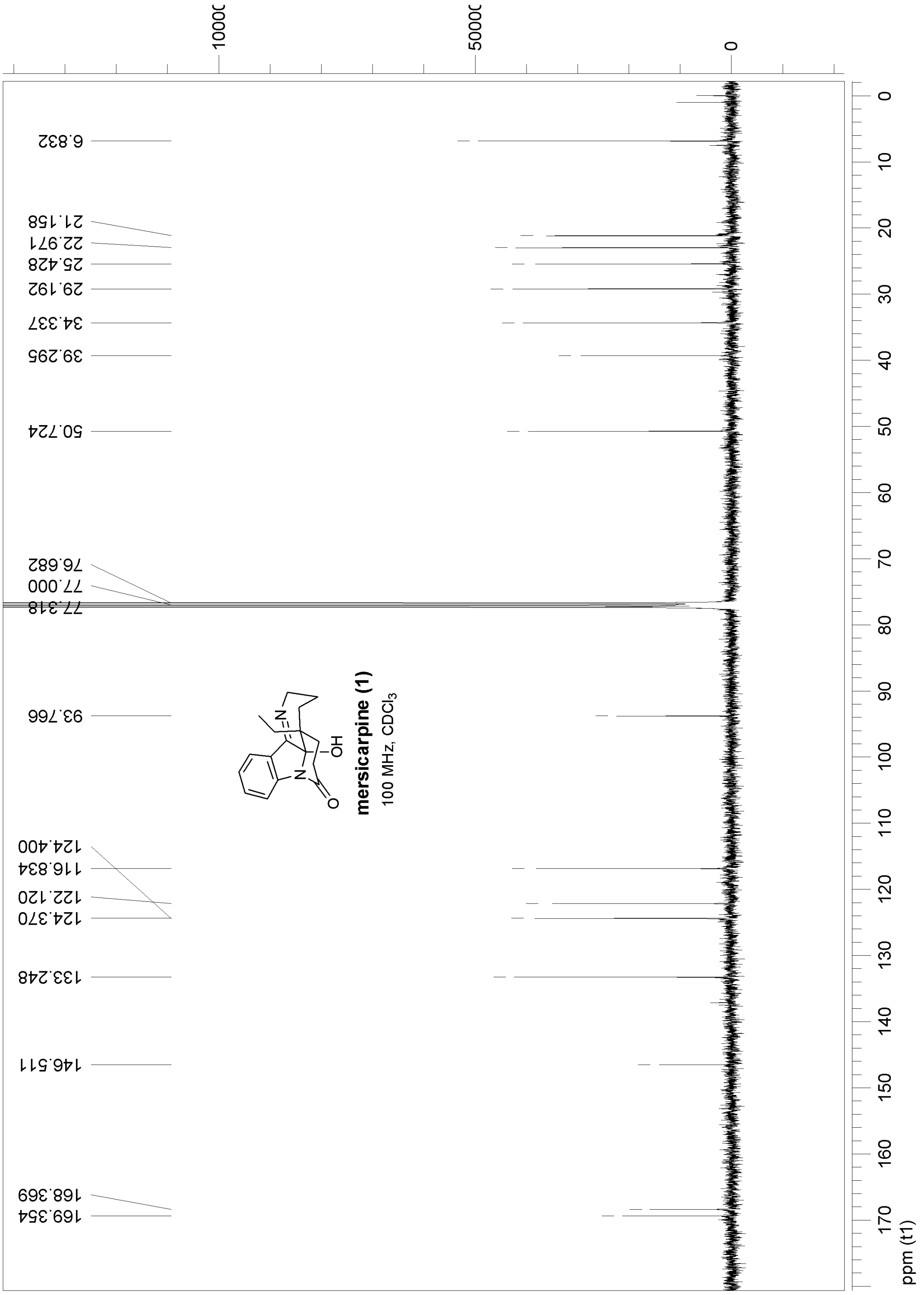

6.832
21.158
22.971
25.428
29.192
34.337
39.295
50.724
76.682
77.000
77.318
93.766
124.400
116.834
122.120
124.370
133.248
146.511
168.369
169.354
mersicarpine (1)
100 MHz, $CDCl_3$
ppm (t1)

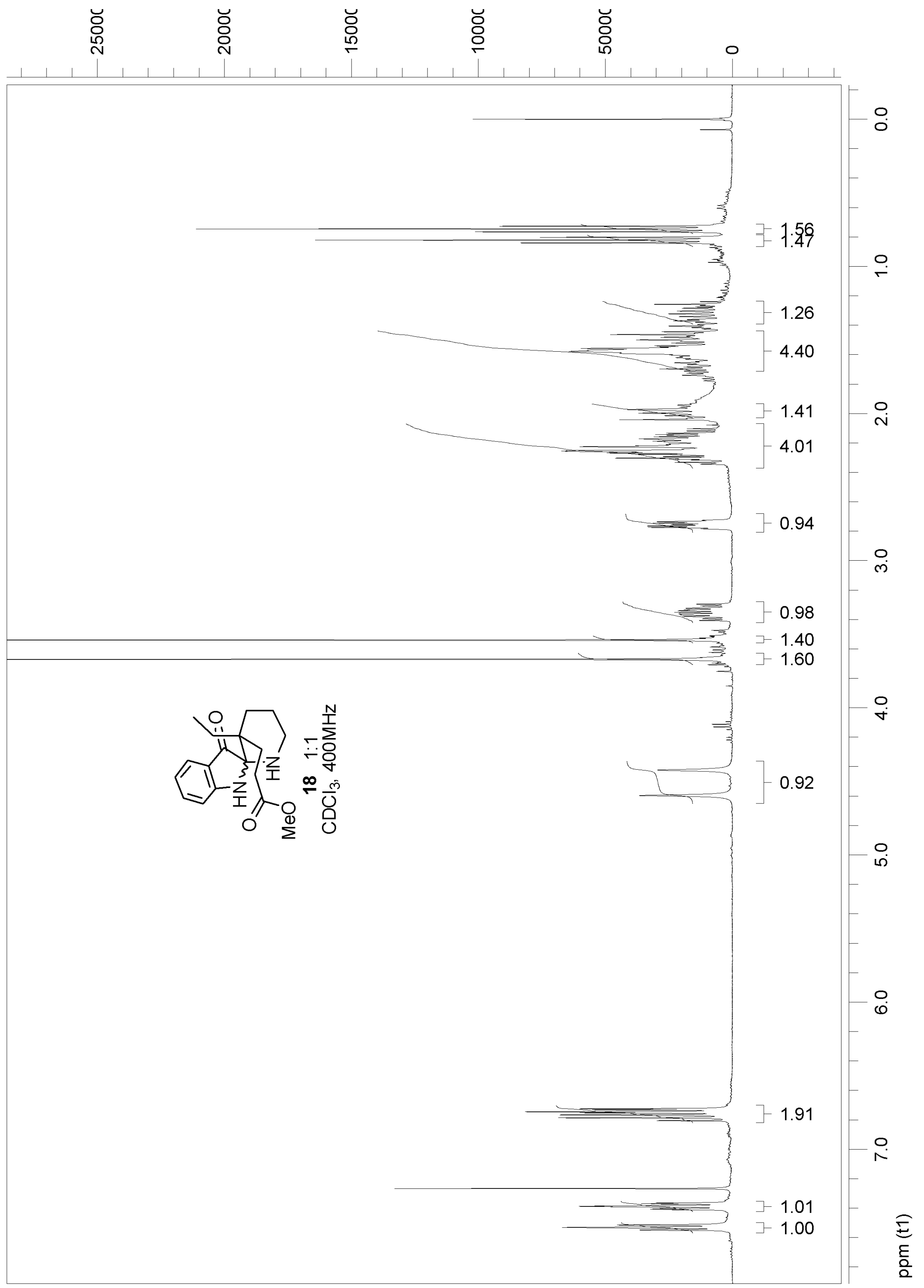
18 1:1
CDCl3, 400MHz
ppm (t1)

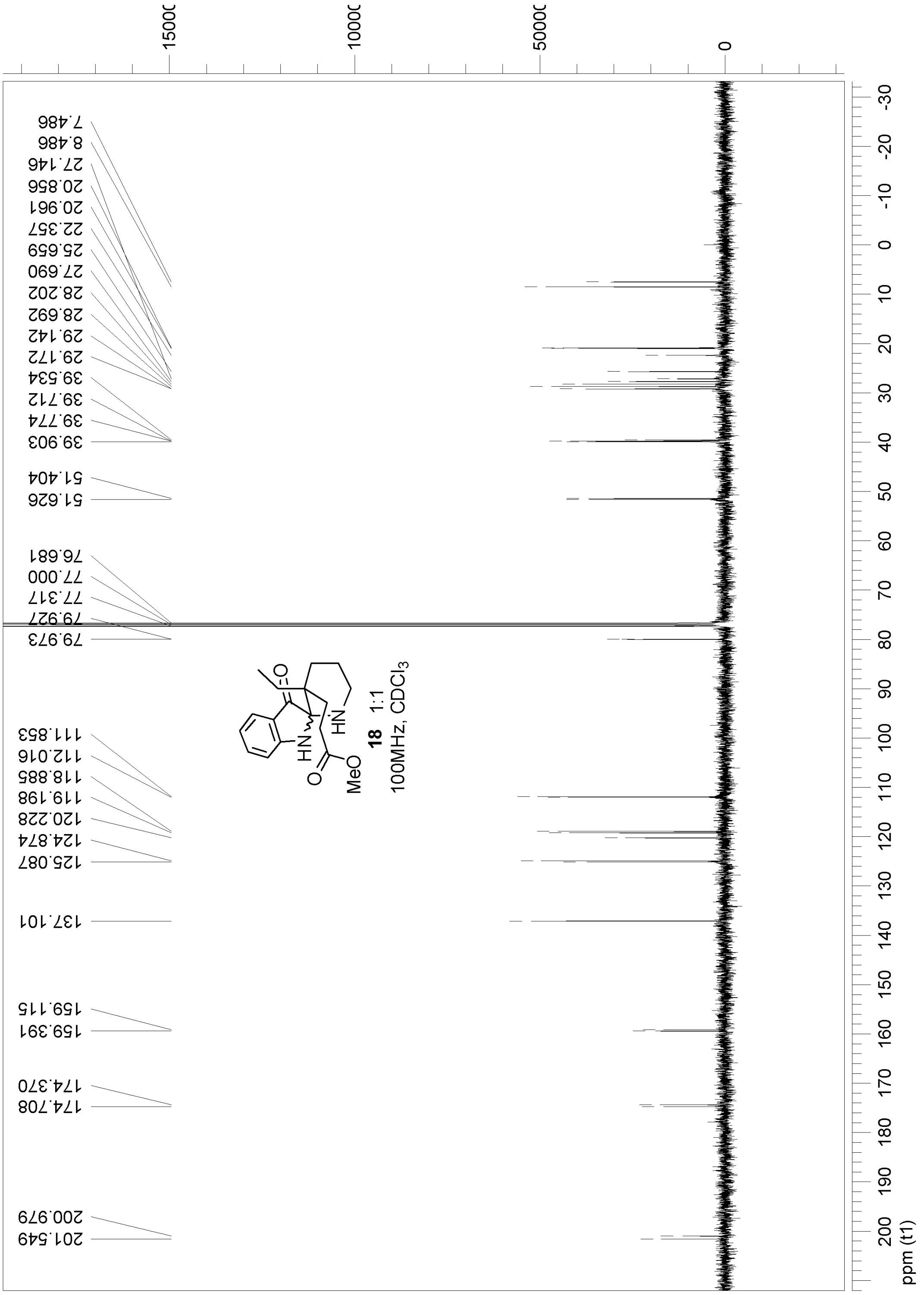
7.486
8.486
27.146
20.856
20.961
22.357
25.659
27.690
28.202
28.692
29.142
29.172
39.534
39.712
39.774
39.903
51.404
51.626
76.681
77.000
77.317
79.927
79.973
111.853
112.016
118.885
119.198
120.228
124.874
125.087
137.101
159.115
159.391
174.370
174.708
200.979
201.549
18 1:1
100MHz, $CDCl_3$
ppm (t1)

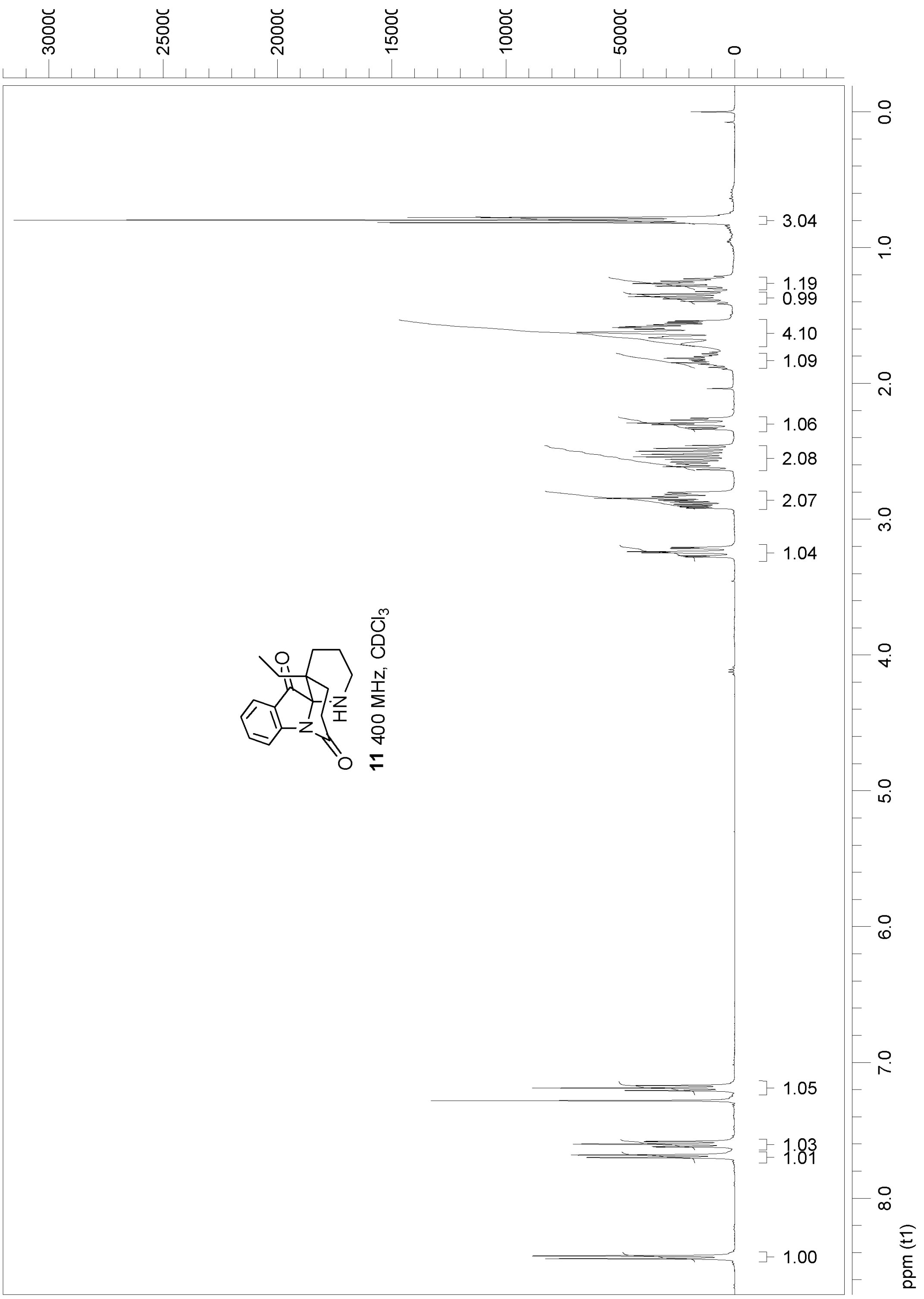

11 400 MHz, CDCl$_3$
ppm (t1)
0.0
1.0
2.0
3.0
4.0
5.0
6.0
7.0
8.0
3.04
1.19
0.99
4.10
1.09
1.06
2.08
2.07
1.04
1.05
1.03
1.01
1.00
30000
25000
20000
15000
10000
5000
0

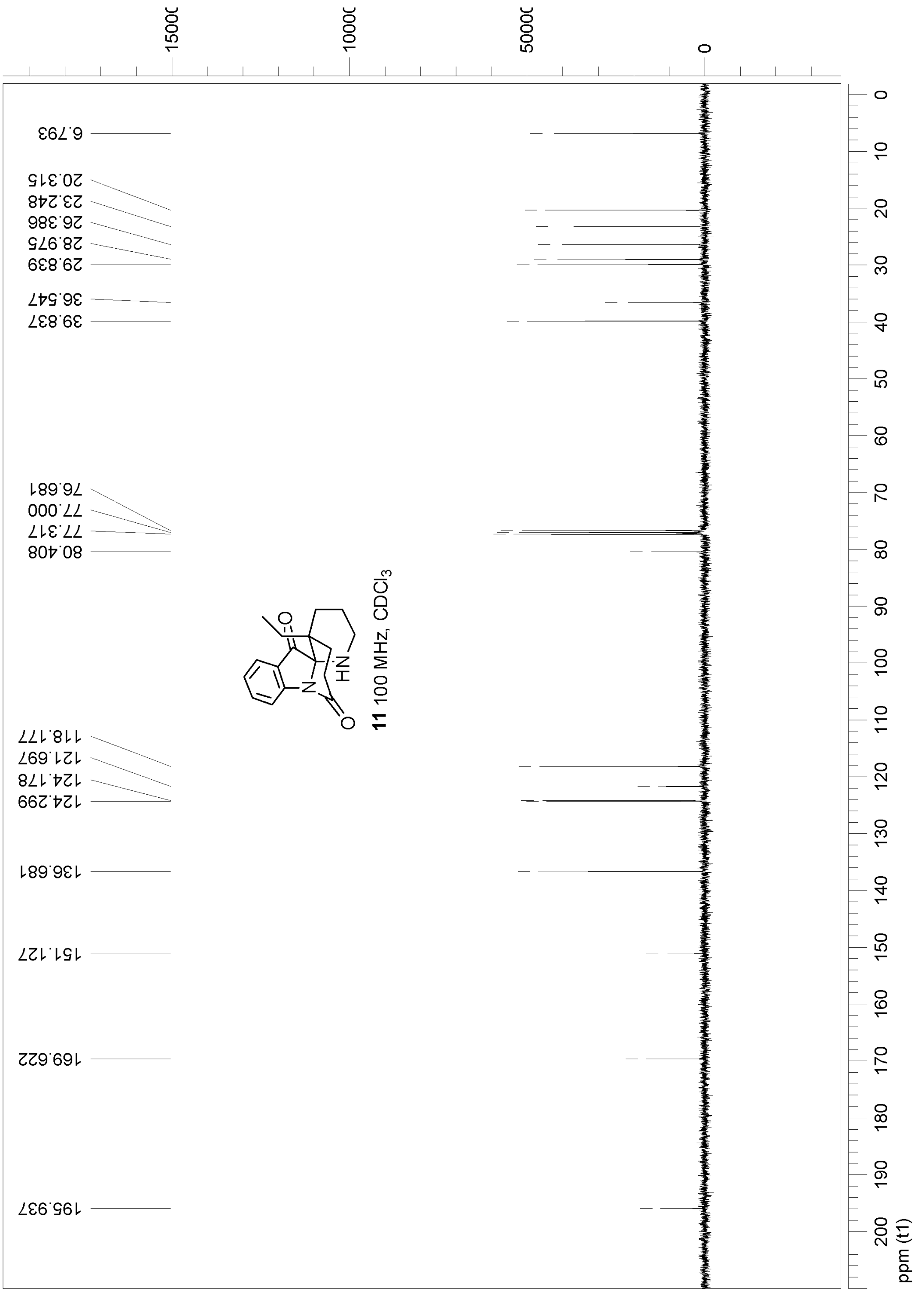
195.937
169.622
151.127
136.681
124.299
124.178
121.697
118.177
80.408
77.317
77.000
76.681
39.837
36.547
29.839
28.975
26.386
23.248
20.315
6.793
11 100 MHz, $CDCl_3$
ppm (t1)

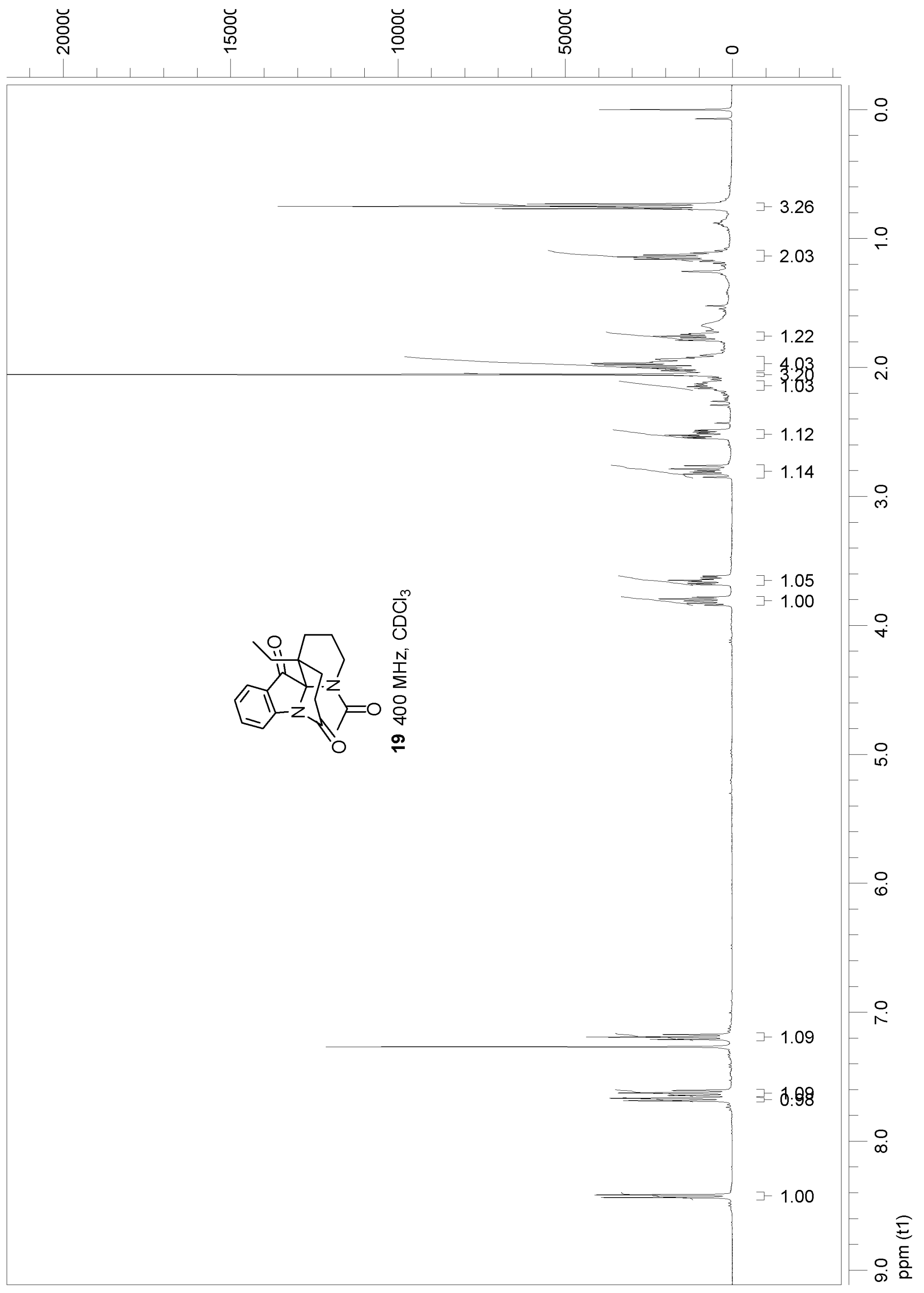
19 400 MHz, CDCl3
ppm (t1)

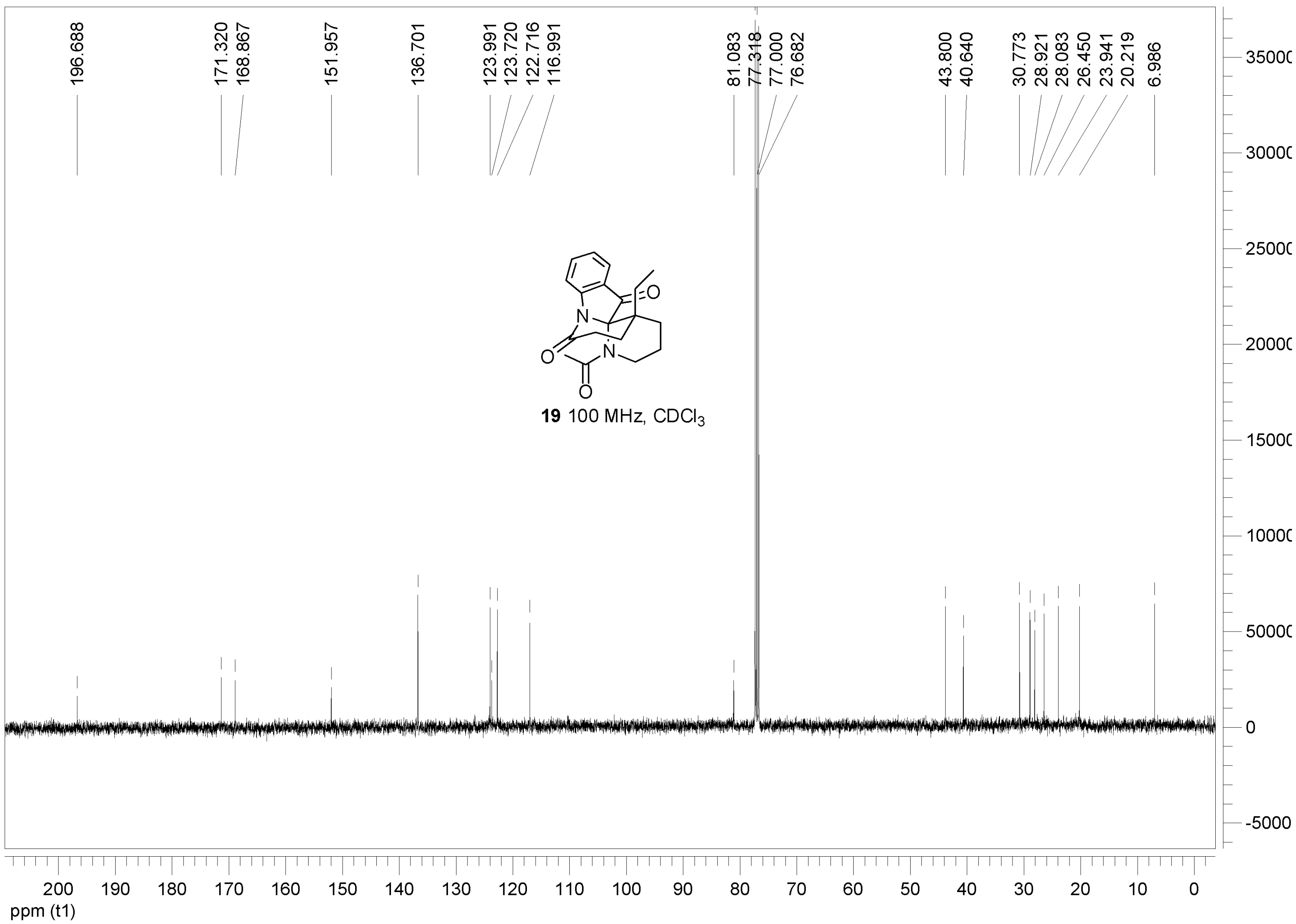

19 100 MHz, $CDCl_3$

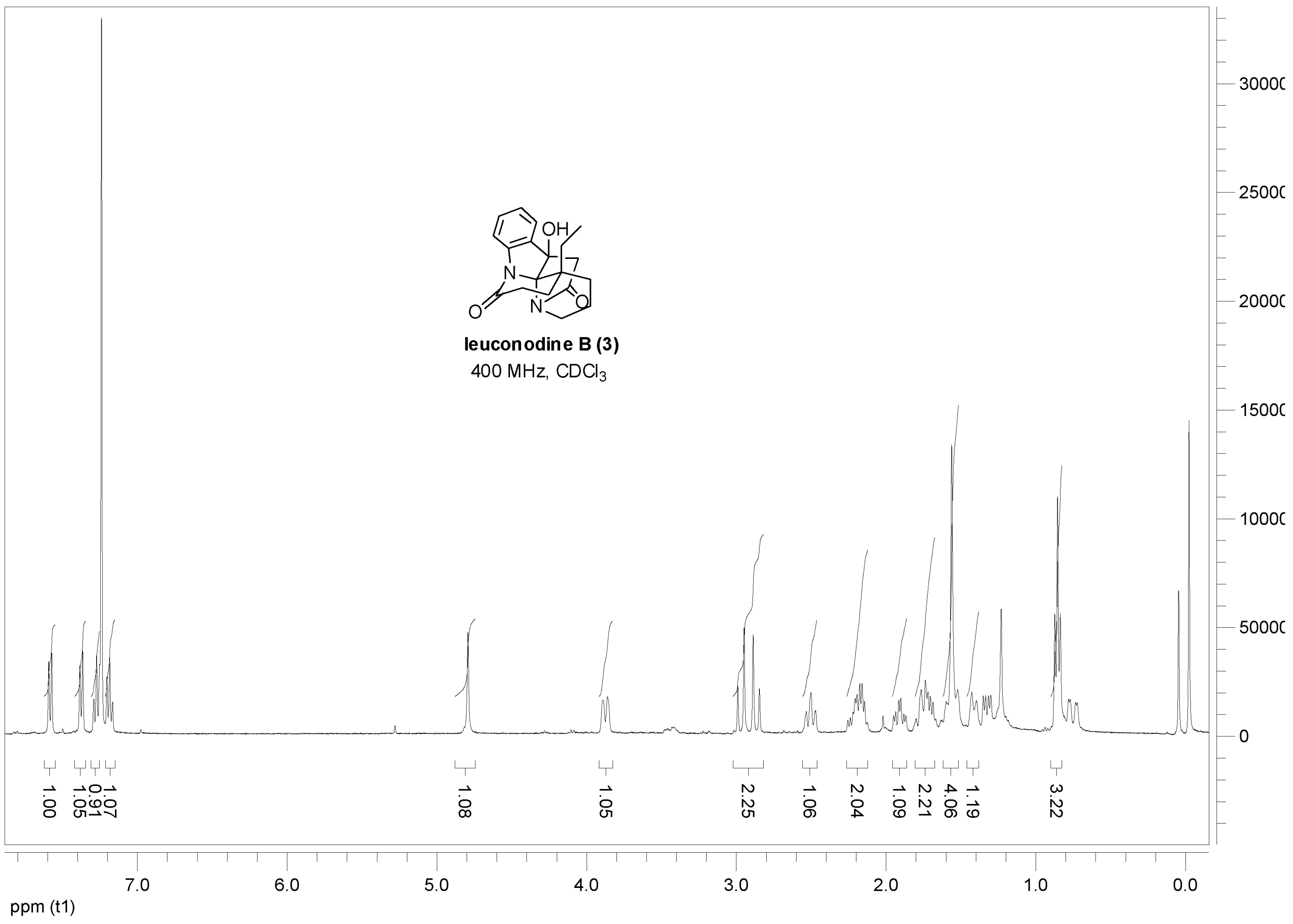
OH
N
N
O
O
leuconodine B (3)
400 MHz, CDCl3
1.00
1.05
0.91
1.07
1.08
1.05
2.25
1.06
2.04
1.09
2.21
4.06
1.19
3.22
7.0
6.0
5.0
4.0
3.0
2.0
1.0
0.0
ppm (t1)
30000
25000
20000
15000
10000
50000
0

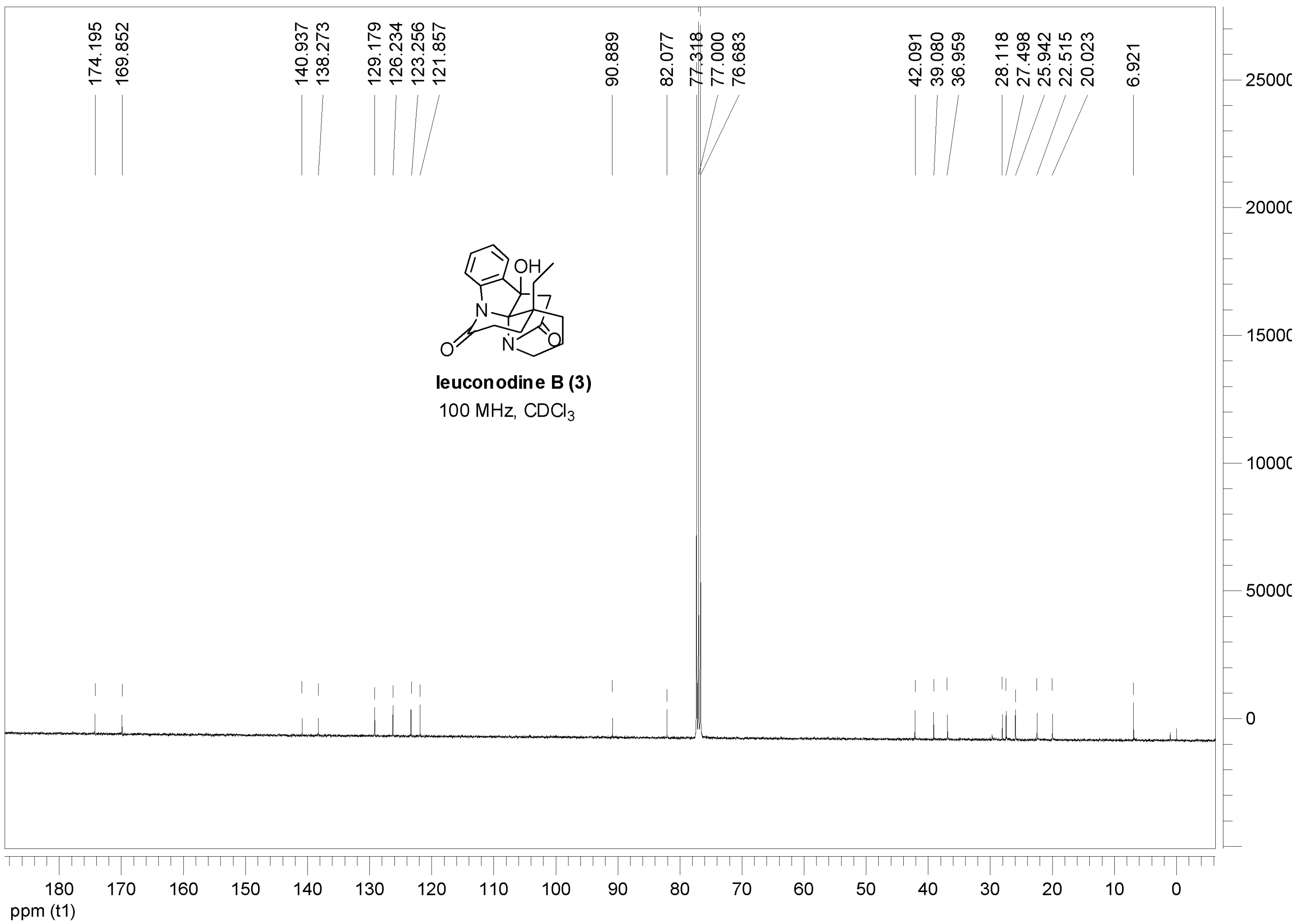
174.195
169.852
140.937
138.273
129.179
126.234
123.256
121.857
90.889
82.077
77.318
77.000
76.683
42.091
39.080
36.959
28.118
27.498
25.942
22.515
20.023
6.921
OH
N
O
leuconodine B (3)
100 MHz, CDCl3
ppm (t1)

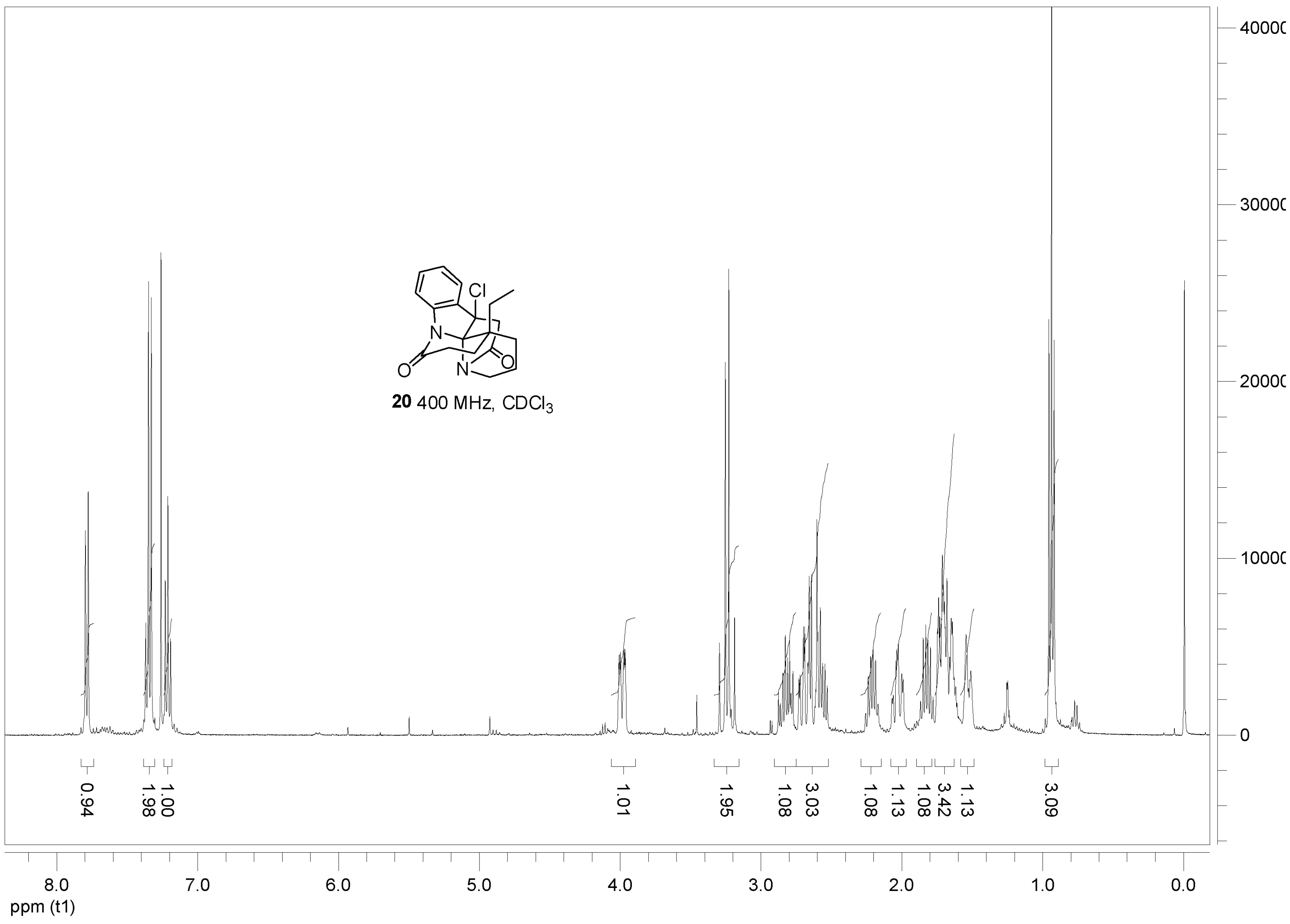

Cl
N
O
N
O
20 400 MHz, CDCl3
40000
30000
20000
10000
0
0.94
1.98
1.00
1.01
1.95
1.08
3.03
1.08
1.13
1.08
3.42
1.13
3.09
8.0
7.0
6.0
5.0
4.0
3.0
2.0
1.0
0.0
ppm (t1)

leuconolam (5)

400 MHz, $CDCl_3$:CD_3OD = 3:1(v:v)

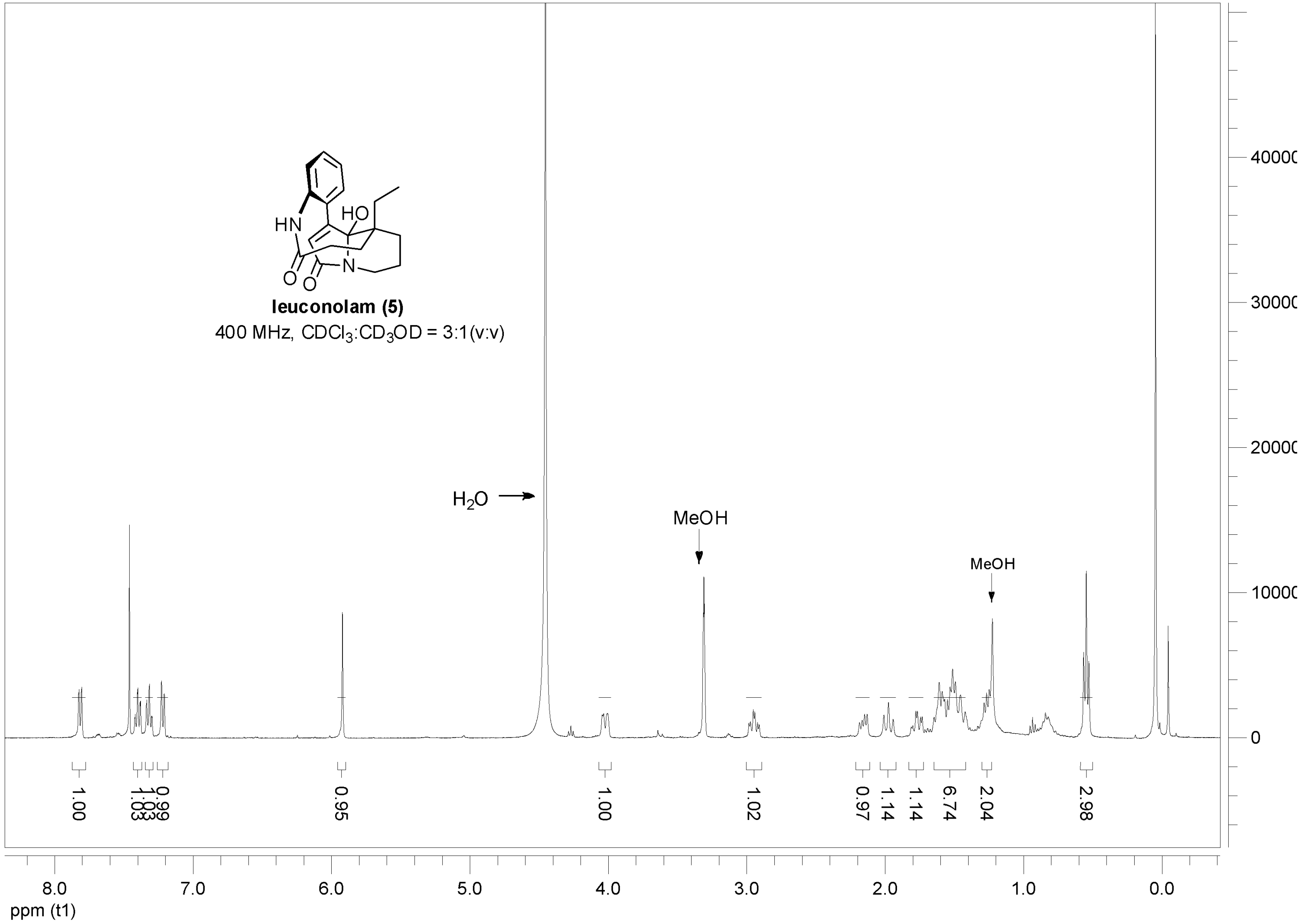

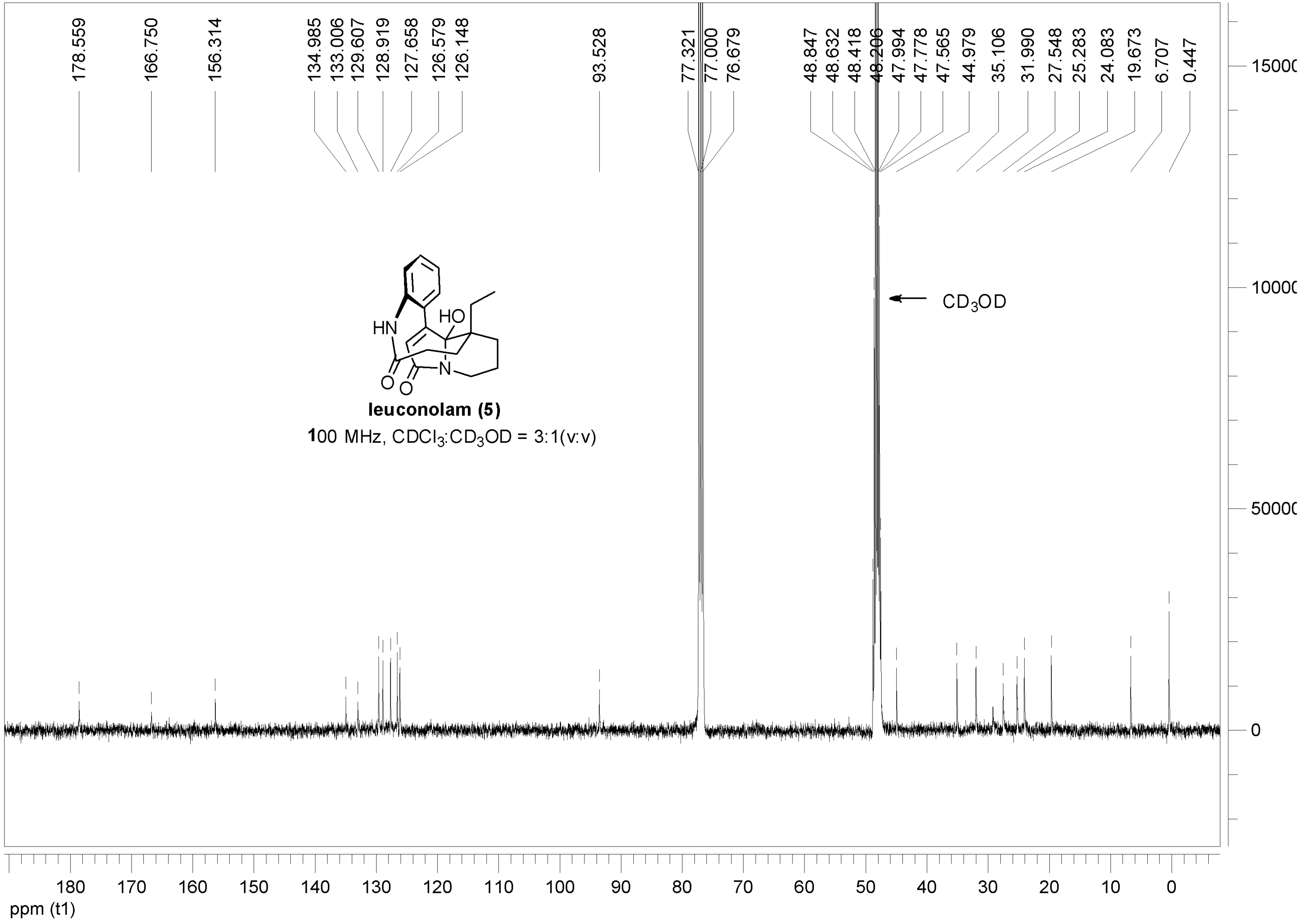
178.559
166.750
156.314
134.985
133.006
129.607
128.919
127.658
126.579
126.148
93.528
77.321
77.000
76.679
48.847
48.632
48.418
48.206
47.994
47.778
47.565
44.979
35.106
31.990
27.548
25.283
24.083
19.673
6.707
0.447
HN
HO
N
O
O
CD_3OD
leuconolam (5)
100 MHz, $CDCl_3$:CD_3OD = 3:1(v:v)
15000
10000
50000
0
180
170
160
150
140
130
120
110
100
90
80
70
60
50
40
30
20
10
0
ppm (t1)

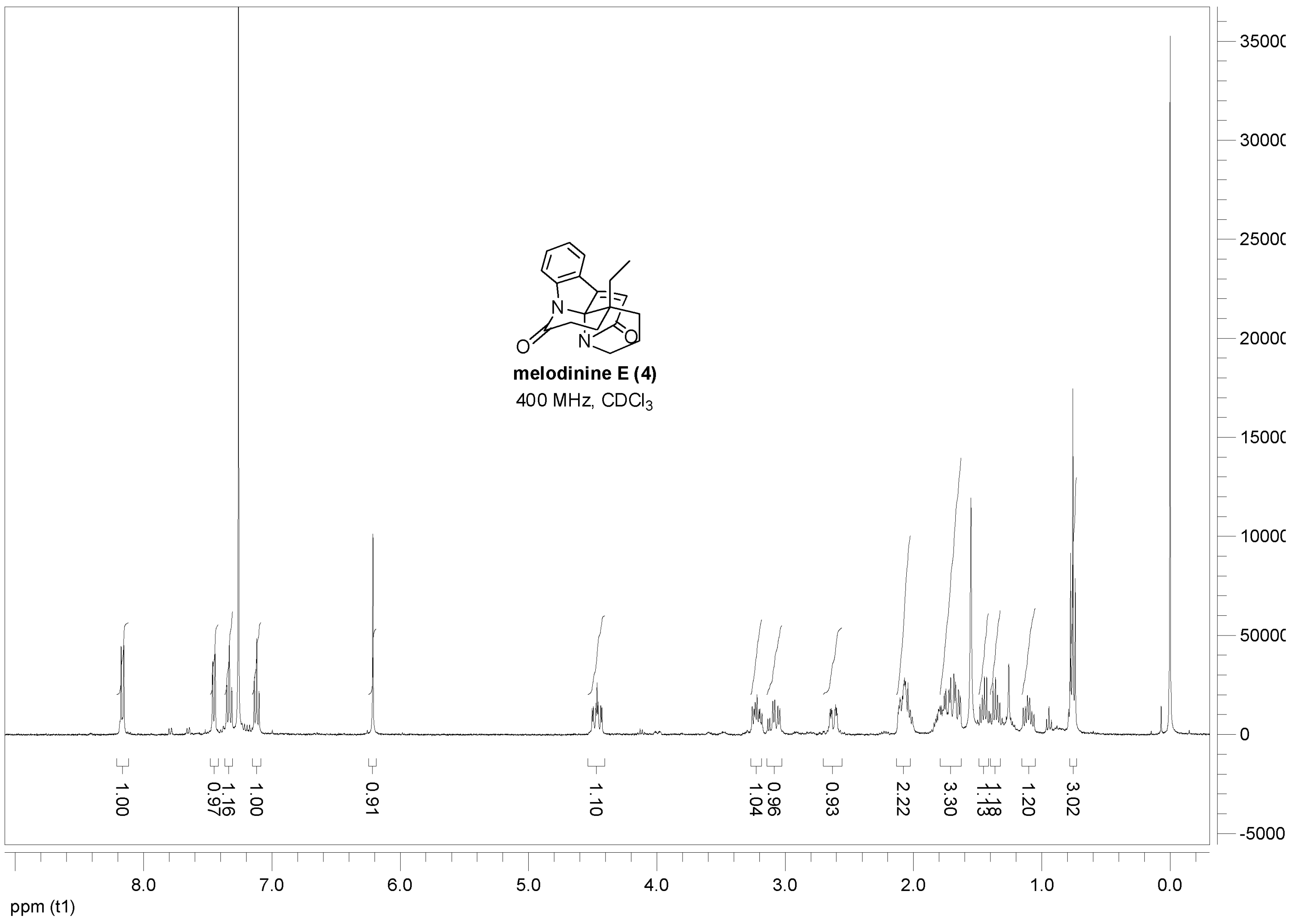

melodinine E (4)
400 MHz, $CDCl_3$
ppm (t1)

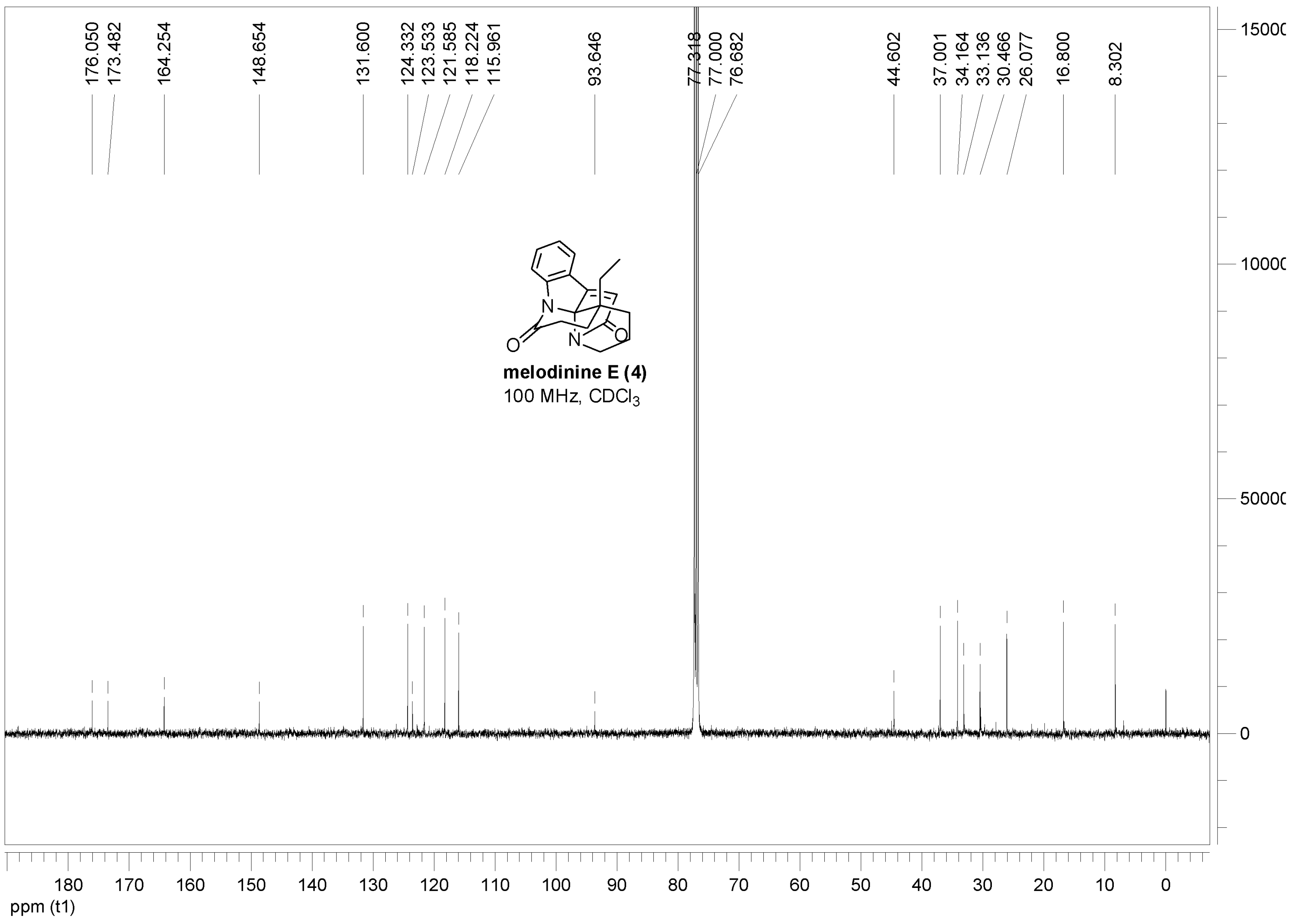
176.050
173.482
164.254
148.654
131.600
124.332
123.533
121.585
118.224
115.961
93.646
77.318
77.000
76.682
44.602
37.001
34.164
33.136
30.466
26.077
16.800
8.302
melodinine E (4)
100 MHz, $CDCl_3$
ppm (t1)

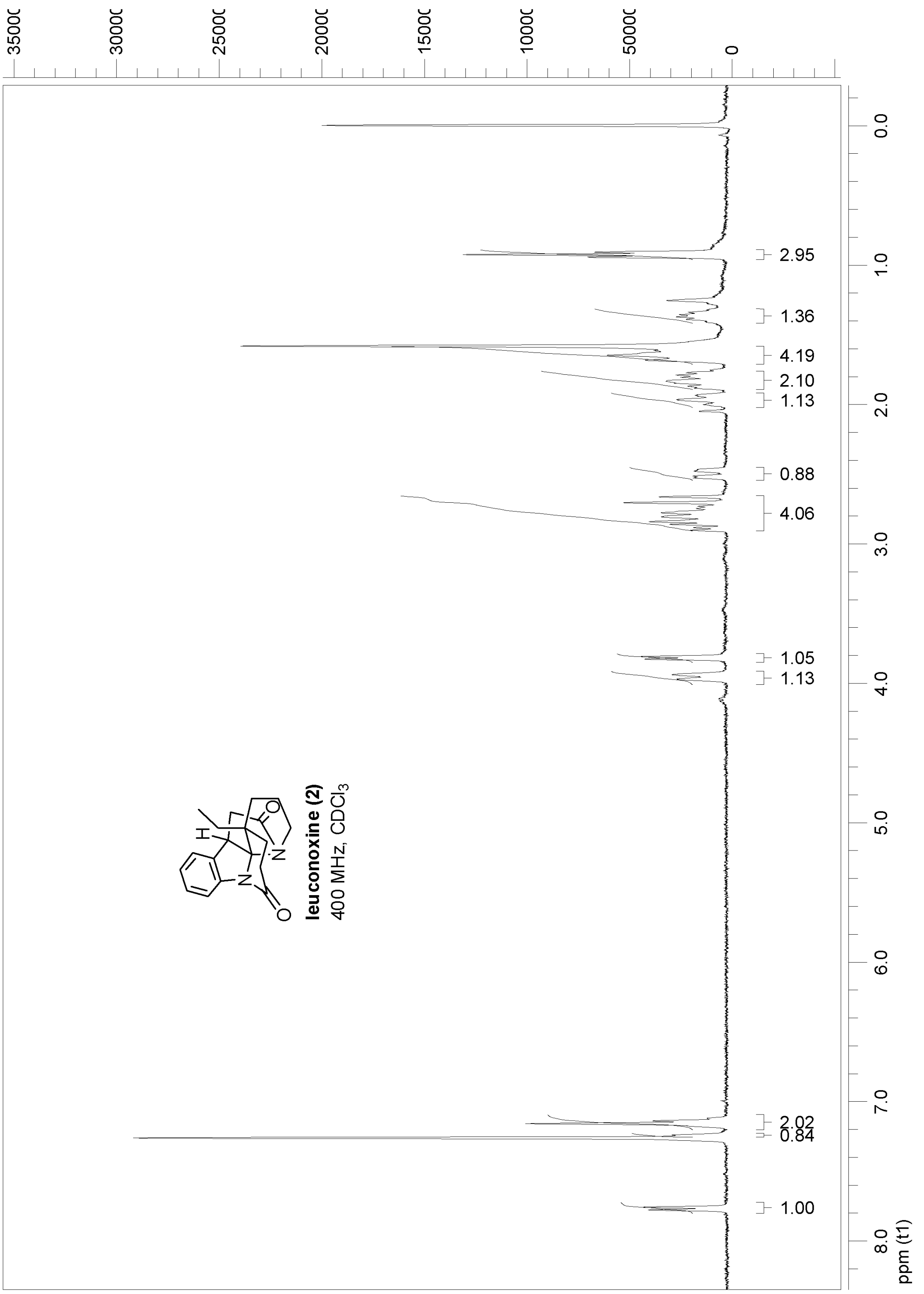
leuconoxine (2)
400 MHz, CDCl3
ppm (t1)
0.0
1.0
2.0
3.0
4.0
5.0
6.0
7.0
8.0
2.95
1.36
4.19
2.10
1.13
0.88
4.06
1.05
1.13
2.02
0.84
1.00
0
5000
10000
15000
20000
25000
30000
35000
N
N
O
O
H

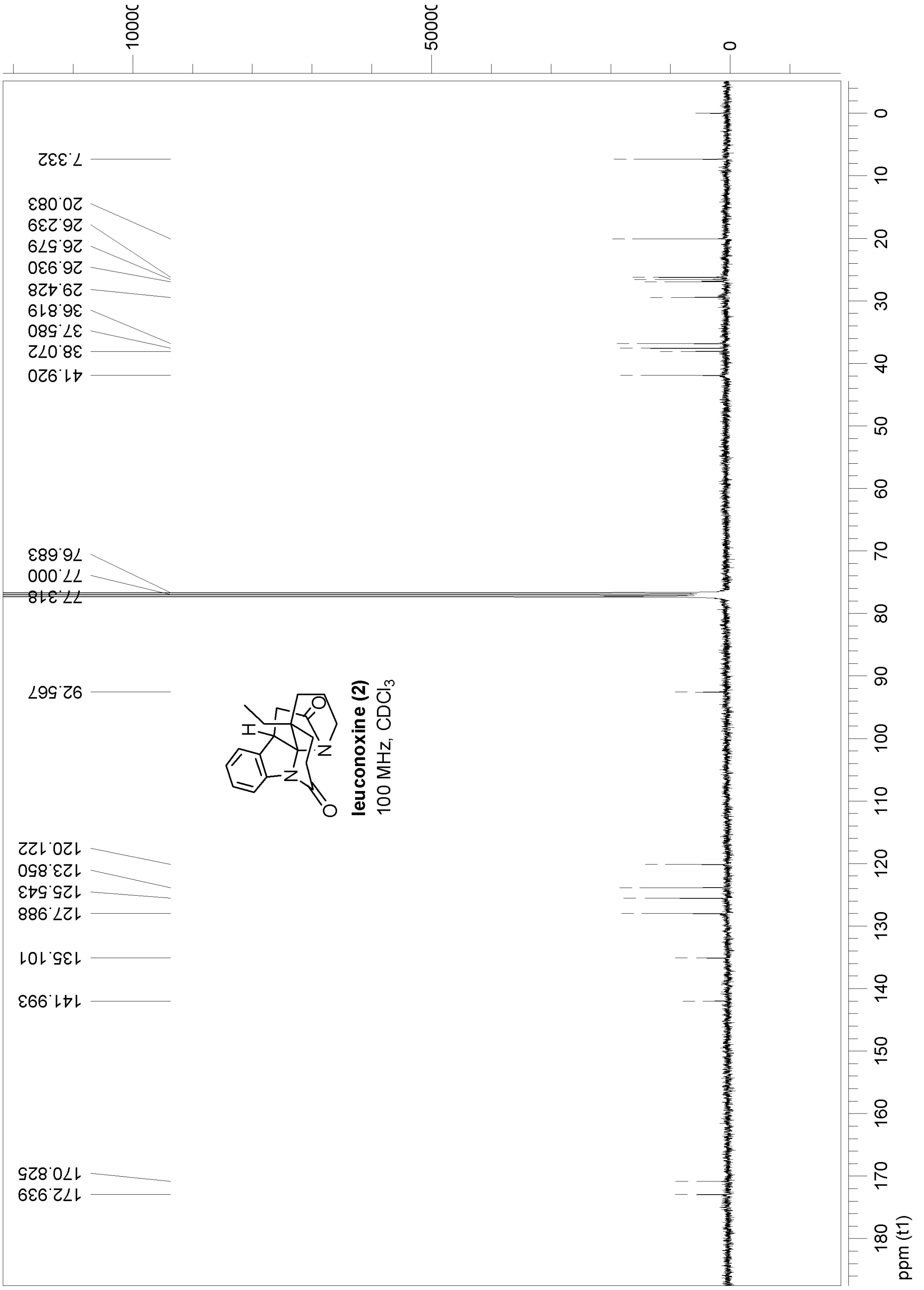
7.332
20.083
26.239
26.579
26.930
29.428
36.819
37.580
38.072
41.920
76.683
77.000
77.318
92.567
120.122
123.850
125.543
127.988
135.101
141.993
170.825
172.939
leuconoxine (2)
100 MHz, CDCl3
ppm (t1)

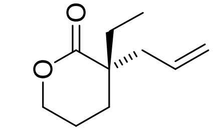

(–)-**12** 500 MHz, $CDCl_3$

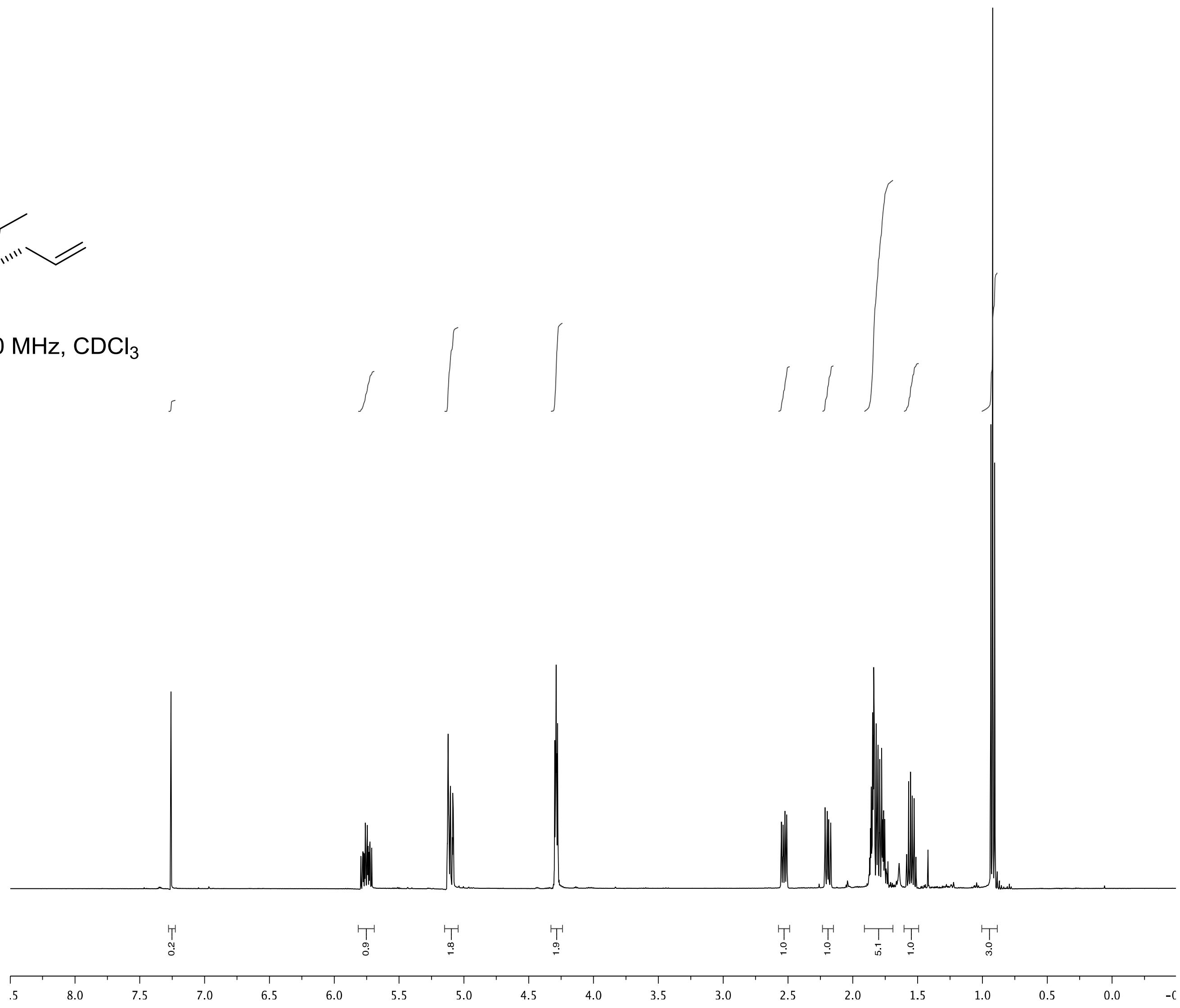

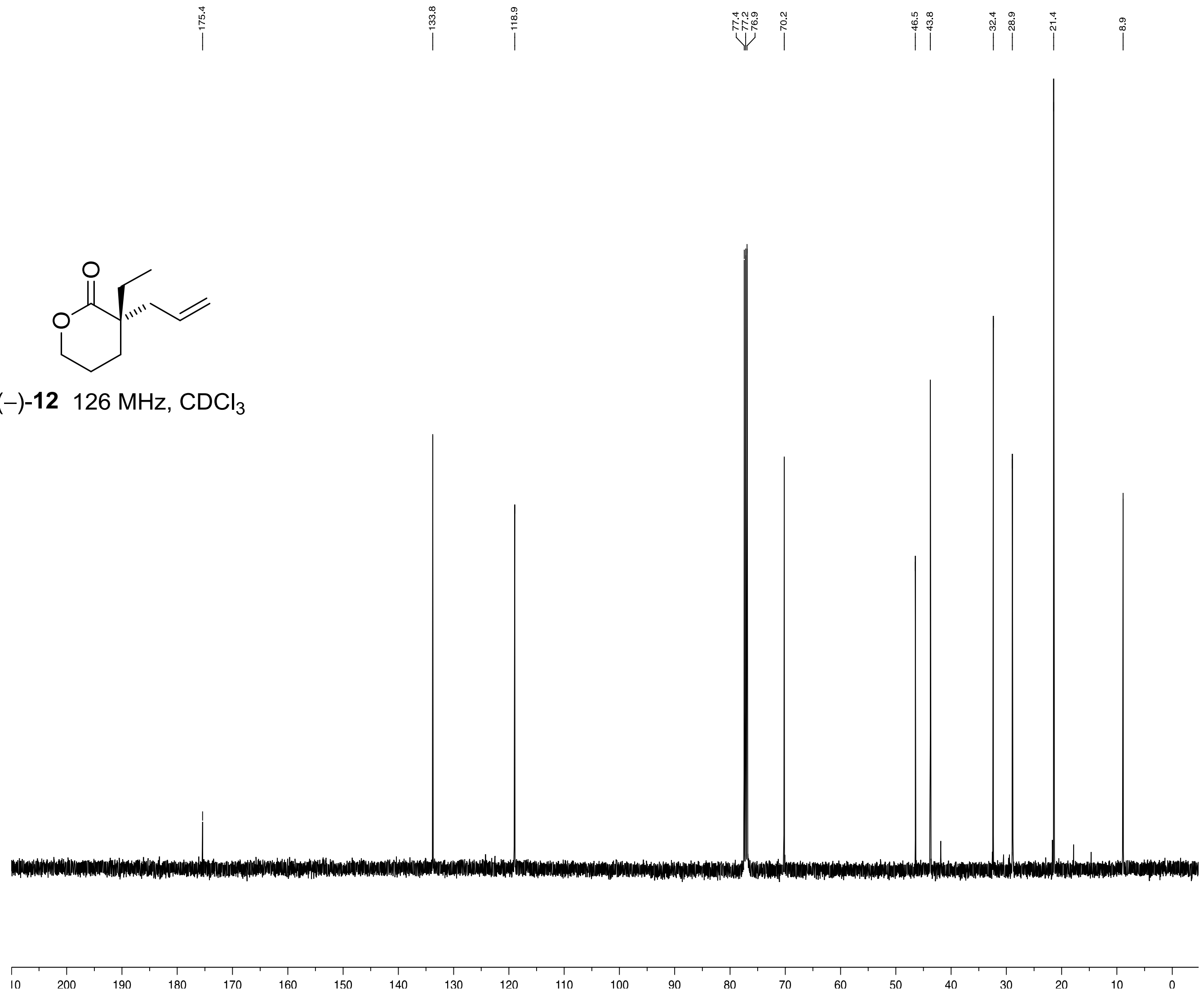

(–)-**12** 126 MHz, $CDCl_3$

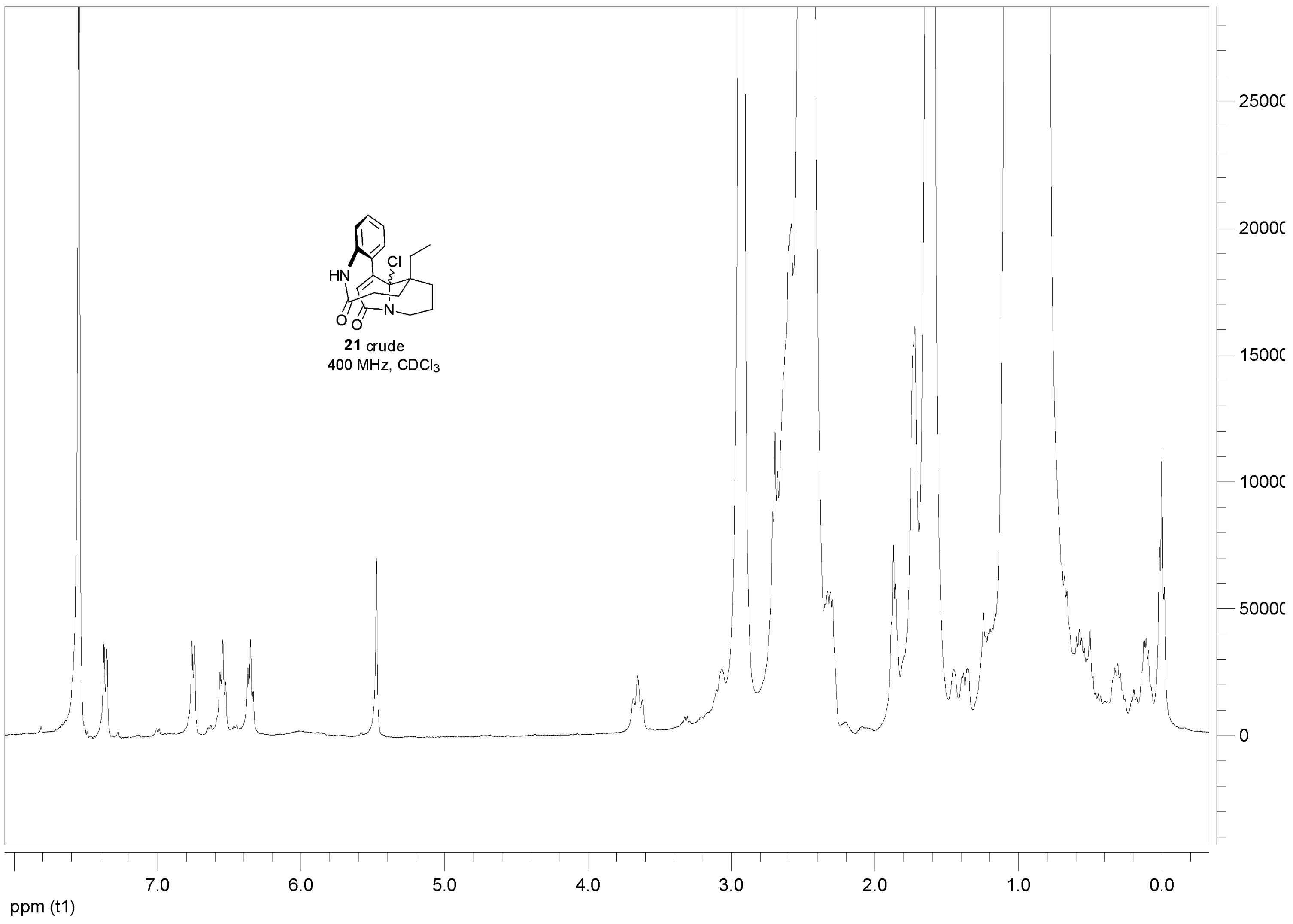

HN
Cl
O
O
N
21 crude
400 MHz, $CDCl_3$
25000
20000
15000
10000
50000
0
7.0
6.0
5.0
4.0
3.0
2.0
1.0
0.0
ppm (t1)

HRMS (ESI) Calcd for $C_{19}H_{22}ClN_2O_2$ $[M+H]^+$ 345.1364 **(21)**

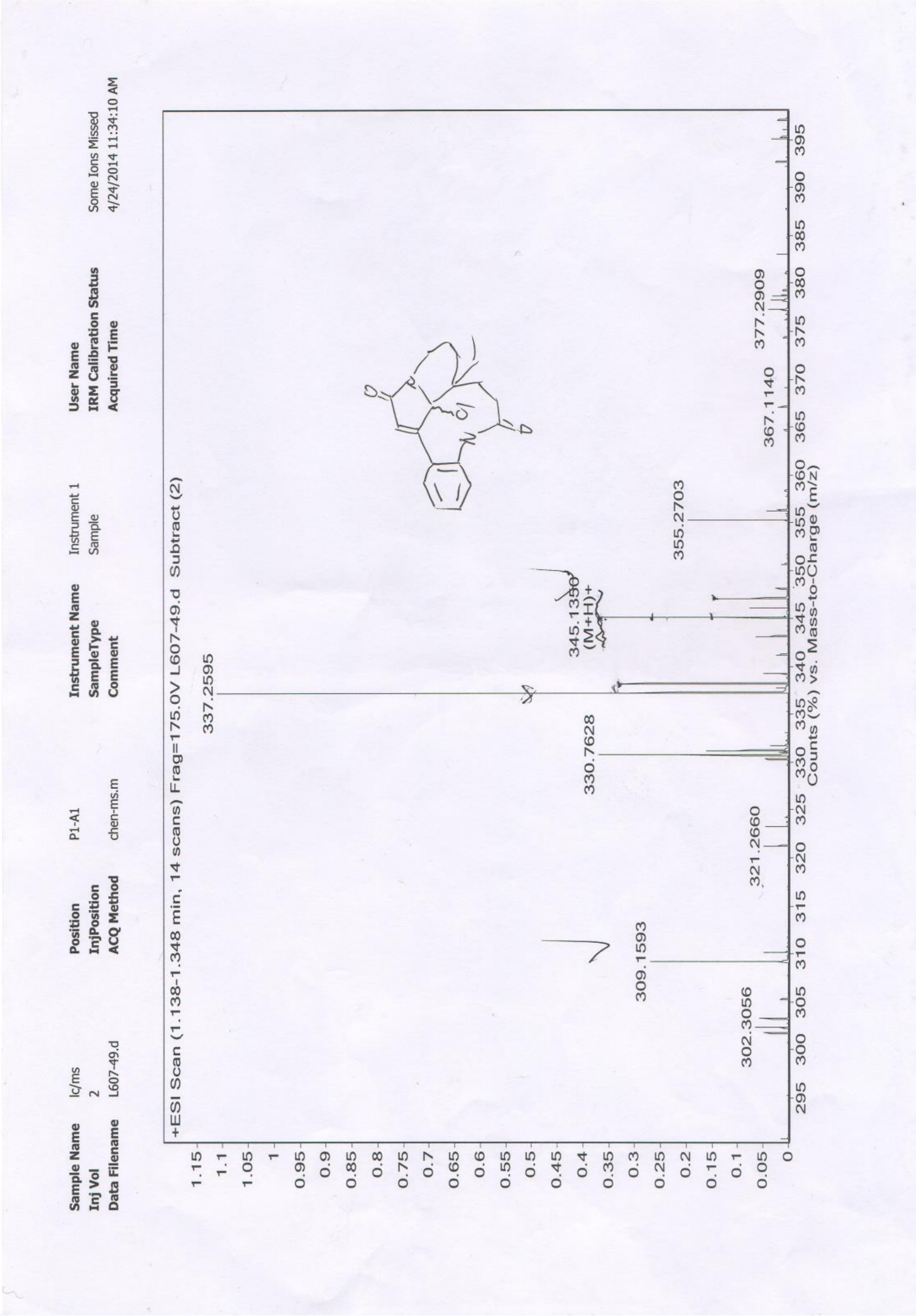

Supplement: Supplementary file 1 [file QO-002-c4qo00312h-s001.pdf]
